# Effect of Casein Hydrolysate on Cardiovascular Risk Factors: A Systematic Review and Meta-Analysis of Randomized Controlled Trials

**DOI:** 10.3390/nu14194207

**Published:** 2022-10-09

**Authors:** Shuaishuai Zhou, Teng Xu, Xu Zhang, Junjie Luo, Peng An, Yongting Luo

**Affiliations:** Department of Nutrition and Health, China Agricultural University, Beijing 100193, China

**Keywords:** blood pressure, blood lipids, cardiovascular disease, casein hydrolysate

## Abstract

Casein hydrolysate has various biological functional activities, especially prominent are angiotensin I-converting enzyme inhibitory activities. Increasing evidence has reported the prominent hypotensive effect of casein hydrolysate. However, the effects of casein hydrolysate on cardiovascular risk factors remain unclear and require more comprehensive and detailed studies. Here, we conducted a systematic review and meta-analysis on eligible randomized controlled trials (RCTs) to summarize the effects of casein hydrolysate supplementation on blood pressure, blood lipids, and blood glucose. In the pooled analyses, casein hydrolysate significantly reduced systolic blood pressure by 3.20 mmHg (−4.53 to −1.87 mmHg) and diastolic blood pressure by 1.50 mmHg (−2.31 to −0.69 mmHg). Supplementation of casein hydrolysate displayed no effect on total cholesterol (−0.07 mmol/L; −0.17 to 0.03 mmol/L), low-density lipoprotein cholesterol (−0.04 mmol/L; −0.15 to 0.08 mmol/L), high-density lipoprotein cholesterol (−0.01 mmol/L; −0.06 to 0.03 mmol/L), triglycerides (−0.05 mmol/L, −0.14 to 0.05 mmol/L), or fasting blood glucose (−0.01 mmol/L; −0.10 to 0.09 mmol/L) compared with the placebo diets. Collectively, this study indicated that supplementation of casein hydrolysate displayed decreasing effect on blood pressure without affecting blood lipids or glycemic status.

## 1. Introduction

With the aging of the world population, cardiovascular disease (CVD) has become an increasingly prominent public health problem globally. CVD remains the leading cause of mortality worldwide [1]. According to World Health Organization’s 2021 World Health Statistics, global CVD deaths had grown by one quarter since 2000, reaching 17.9 million by 2019 [2]. More seriously, CVD deaths are expected to reach 22.2 million by 2030 [3].

Nutritional and lifestyle risk factors such as suboptimal diet, smoking, alcohol consumption, physical inactivity, and other metabolic diseases (e.g., type 2 diabetes and obesity) were identified to be major risk factors for CVDs [4], among which a healthy diet has been recommended as a preventive or treatment approach for CVDs [5]. Although a variety of foods with cardioprotective potentials have been identified by epidemiological studies [6], the optimal nutrient composition for CVD remains to be investigated [7].

Evidence from randomized controlled trials (RCTs) indicated that milk protein supplementation was an effective diet approach for reducing blood pressure (BP) [8,9] specifically identifying casein as the most abundant high-quality protein in milk, which accounts for 80% of total milk protein [10]. Casein contains nearly all common amino acids and most essential amino acids [11]. It belongs to a slow-digesting protein, possessing a delayed release property in the digestive system. In addition, it also has anti-catabolic properties and inhibits the breakdown of other proteins [12]. Through commercial fermentation and enzymatic hydrolysis, casein can be converted into casein hydrolysate and other active peptides [13].

Casein-derived hydrolysate has various biological functions including, but not limited to, anti-inflammatory, antioxidant [14], and antihypertensive activities [15,16]. Oxidative stress and chronic inflammation play critical roles in the process of CVD development [17,18]. Hence, it is essential to demonstrate how casein hydrolysate regulates cardiometabolic health. Strong evidence indicates that casein hydrolysate lowers BP by blocking the activity of angiotensin I-converting enzyme (ACE) [10,16,19], which converts angiotensin I to vasoconstrictor angiotensin II by removing two amino acids from the C-terminus of the active peptides [20]. In addition to BP elevation, angiotensin II can stimulate the release of pro-inflammatory cytokines and the activation of nuclear factor kappa B (NFkB) [21,22]. The antioxidant properties of casein hydrolysate might be attributed to its powerful scavenging capacity of superoxides and hydroxyl radicals, as well as its chelating activity on iron ions [23,24,25].

Contradictory results have been reported on the BP lowering effect of casein hydrolysate in RCTs [26,27]. Therefore, systematic reviews and meta-analyses were performed to assess the effects of casein hydrolysate supplementation on BP [28,29,30,31]. However, these published systematic reviews and meta-analyses need updating to incorporate recent published studies. In addition, the influences of casein-derived hydrolysate on CVD risk factors also require a systematical assessment. To provide the most updated and comprehensive evaluation, we conducted a systematic review and meta-analysis of published RCTs to assess the effects of casein hydrolysate on multiple CVD risk factors, including BP, blood lipids, and blood glucose.

## 2. Methods

The systematic review and meta-analysis were performed according to the Preferred Reporting Items for Systematic Reviews and Meta-Analyses (PRISMA) guidelines.

### 2.1. Search Strategy

We searched the PubMed and Web of Science databases from 1994 to July 2022 to identify eligible studies. The involved searching keywords were randomized controlled trial, casein hydrolysate, cardiovascular disease, systolic blood pressure (SBP), diastolic blood pressure (DBP), total cholesterol (TC), low-density lipoprotein cholesterol (LDL-C), high-density lipoprotein cholesterol (HDL-C), triglycerides (TG), and fasting blood glucose (FBG). Two reviewers independently searched and assessed the studies retrieved from databases. Any inconsistency was reexamined by another reviewer and resolved via group discussion.

### 2.2. Study Selection and Selection Criteria

Inclusion criteria were as follows: (1) the study was written in the English language; (2) trials were performed with humans; (3) published RCTs; (4) the intervention diet was casein hydrolysate or lactotripeptides; (5) the study contained a comparable placebo or control group; (6) and reporting at least one CVD risk factor. Exclusion criteria were as follows: (1) the trials involved animals (e.g., rats or mice); (2) no control/placebo group; (3) the intervention diet was not casein hydrolysate or lactotripeptides; (4) no relevant result of CVD risk factor; (5) no result of intervention or control/placebo group; (6) review article. Initially, two reviewers independently screened the retrieved articles based on titles and abstracts to determine whether the studies met the inclusion criteria. The full text was downloaded for detailed examination for eligible or uncertain studies according to titles and abstracts.

### 2.3. Data Extraction

The following information of eligible studies was extracted: (1) author name; (2) publication year; (3) the study design (randomization, single-or double-blinded; parallel trial, or crossover trial); (4) the source of hydrolysate consumed in the intervention diet; (5) the preparation method of hydrolysate; (6) intervention diet; (7) control/placebo diet; (8) outcomes of CVD risk factor before and after control and intervention diet; (9) intervention duration; (10) health status of participants; (11) population size; (12) geographic location; (13) mean age of participants. For subsequent statistical analysis, the units of TG, related cholesterol indicators, and FBG were unified. The TG value (in mg/dL) was divided by 88.57 (in mmol/L). The related cholesterol value (in mg/dL) was divided by 38.67 (in mmol/L). The FBG value (in mg/dL) was divided by 18 (in mmol/L).

### 2.4. Risk of Bias

The risk of bias was assessed by two independent reviewers according to the Cochrane Collaboration Handbook recommendations [32]. Review Manager 5.4 was used to evaluate the risk of bias in seven domains. Seven domains were (1) random sequence generation (selection bias); (2) allocation concealment (selection bias); (3) blinding of participants and personnel (performance bias); (4) blinding of outcome assessment (detection bias); (5) incomplete outcome data (attrition bias); (6) selective reporting (reporting bias); (7) other bias. Every domain of risk of bias was categorized as unclear risk (represented by a yellow circle), high risk (represented by a red circle), or low risk (represented by a green circle). Disagreements were resolved by discussion until a consensus was reached.

### 2.5. Statistical Analysis

The change value of indicators (endpoint minus baseline) used in the analysis were presented as means and standard deviations (SDs). A random-effects model was applied to assess the effect sizes. The evaluated effect sizes were presented as weighted mean difference (WMD) and 95% confidence intervals (CIs) according to the Cochrane Handbook [32]. Meta-regression was conducted to analyze the possible source of heterogeneity [33]: (1) baseline BP (SBP ≥ 140 mmHg and SBP < 140 mmHg; DBP ≥ 90 mmHg and DBP < 90 mmHg); (2) age (≥50 years and <50 years); (3) duration of the intervention (≥8 weeks and <8 weeks); (4) preparation method (fermentation and enzyme); (5) disease (BP disease and healthy). Egger’s linear regression test was performed to evaluate the existence of publication bias (publication bias was regarded to exist when *p* < 0.05). The symmetry of funnel plots was also visually judged in evaluating publication bias. Sensitivity analysis was used to assess the existence of a small study effect and the reliability of the results. Review Manager 5.4 was used to evaluate the effects of casein hydrolysate on CVD risk factors. It also showed the risk of bias and visual funnel plots of the included studies. Review Manager 5.4 was used to generate a forest plot. Stata 15 was used to perform Egger’s linear regression test and sensitivity analysis. *p* < 0.05 was considered as a statistically significant difference.

## 3. Results

### 3.1. Study Selection and Study Characteristics

**Figure 1:**
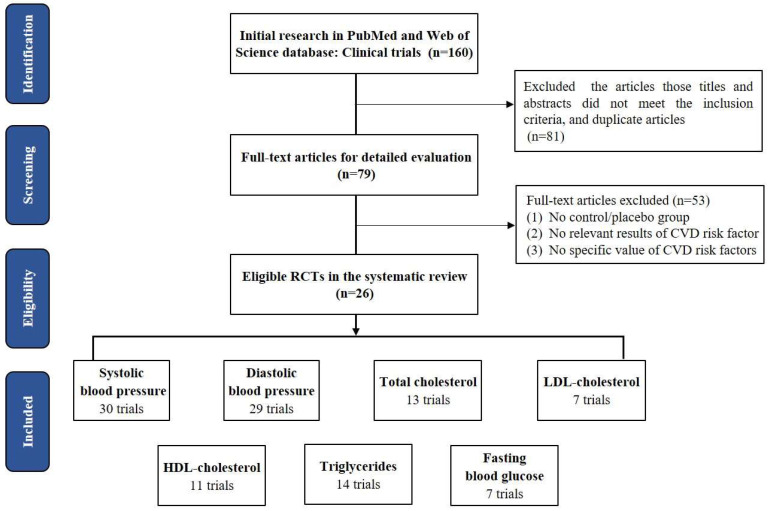
Screening process for eligible studies.

The screening process for eligible studies is shown in Figure 1. We initially retrieved 160 articles in PubMed and Web of Science using fixed search terms (see Supplemental Material). Based on titles and abstracts, 81 articles were removed for duplication or because they did not meet the inclusion criteria. Then, the full texts of the remaining 79 articles were downloaded for detailed evaluation. Based on a full-text review, 53 articles were excluded for no control/placebo group, no relevant results of CVD risk factors, or no specific value of CVD risk factors. Eventually, 26 articles containing 33 trials were included.

In total, 30 trials consisting of 1824 participants reported SBP; 29 trials consisting of 1743 participants reported DBP; 13 trials consisting of 648 participants reported TC; 7 trials consisting of 386 participants reported LDL-C; 11 trials consisting of 641 participants reported HDL-C levels; 14 studies trials consisting of 678 participants reported TG; and 7 trials consisting of 396 participants reported FBG. The detailed characteristics of included studies are described in Table S1. The effects of casein hydrolysate on cardiovascular risk factors (SBP, DBP, TC, LDL, HDL, TG, and FBG) were assessed using a random-effects model.

### 3.2. Effects of Casein Hydrolysate on BP

**Figure 2:**
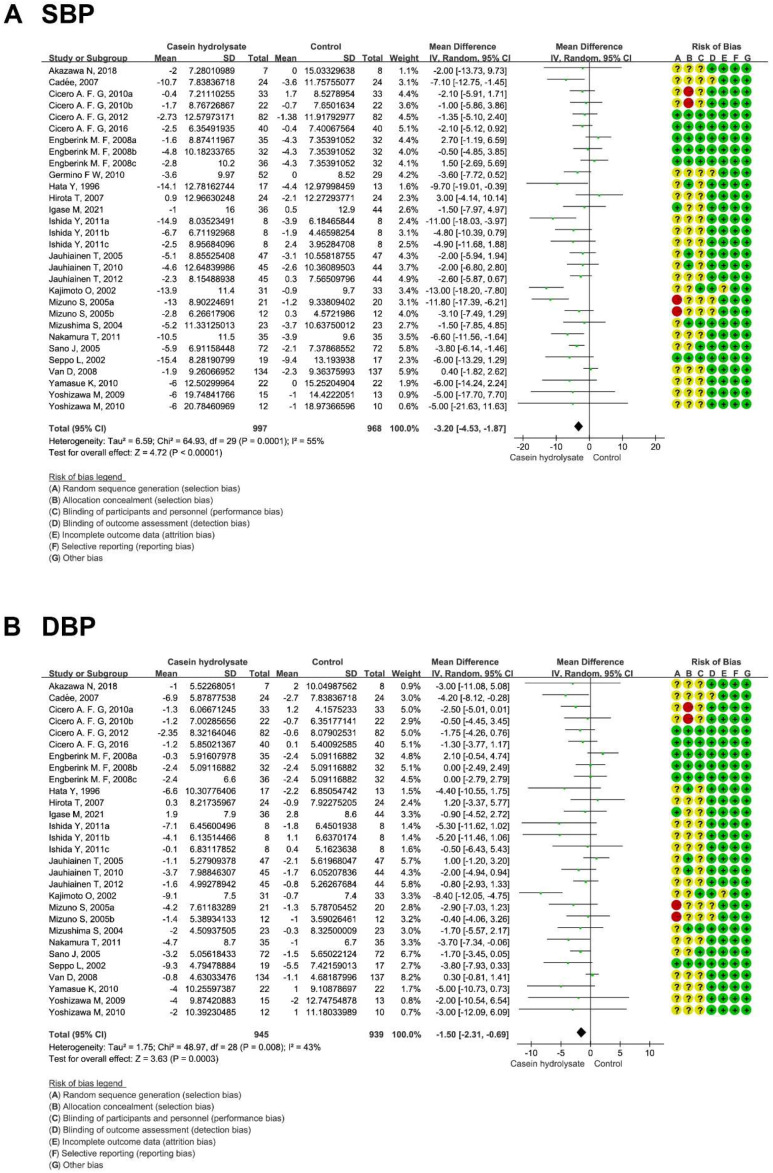
Effect of casein hydrolysate supplementation on blood pressure: (**A**) SBP and (**B**) DBP. Each domain of risk of bias is represented by a circle: a green circle for low risk, a yellow circle for unclear risk, and a red circle for high risk.

Casein hydrolysate significantly reduced SBP by 3.20 mmHg (95% CI: −4.53, −1.87 mmHg; *p* < 0.00001) compared with control diets (Figure 2A). Publication bias was suggested from the visual funnel plot and Egger’s linear regression test (*p* = 0.01) (Figure S1, Table S2). However, sensitivity analysis indicated that the result of SBP was reliable (Figure S2).

Casein hydrolysate supplementation decreased DBP by 1.50 mmHg (95% CI: −2.31, −0.69 mmHg; *p* = 0.0003) (Figure 2B). Publication of bias was suggested by Egger’s linear regression test (*p* = 0.03) (Table S2). Sensitivity analysis indicated that the DBP result was reliable (Figure S2).

### 3.3. Effects of Casein Hydrolysate on Blood Lipids

**Figure 3:**
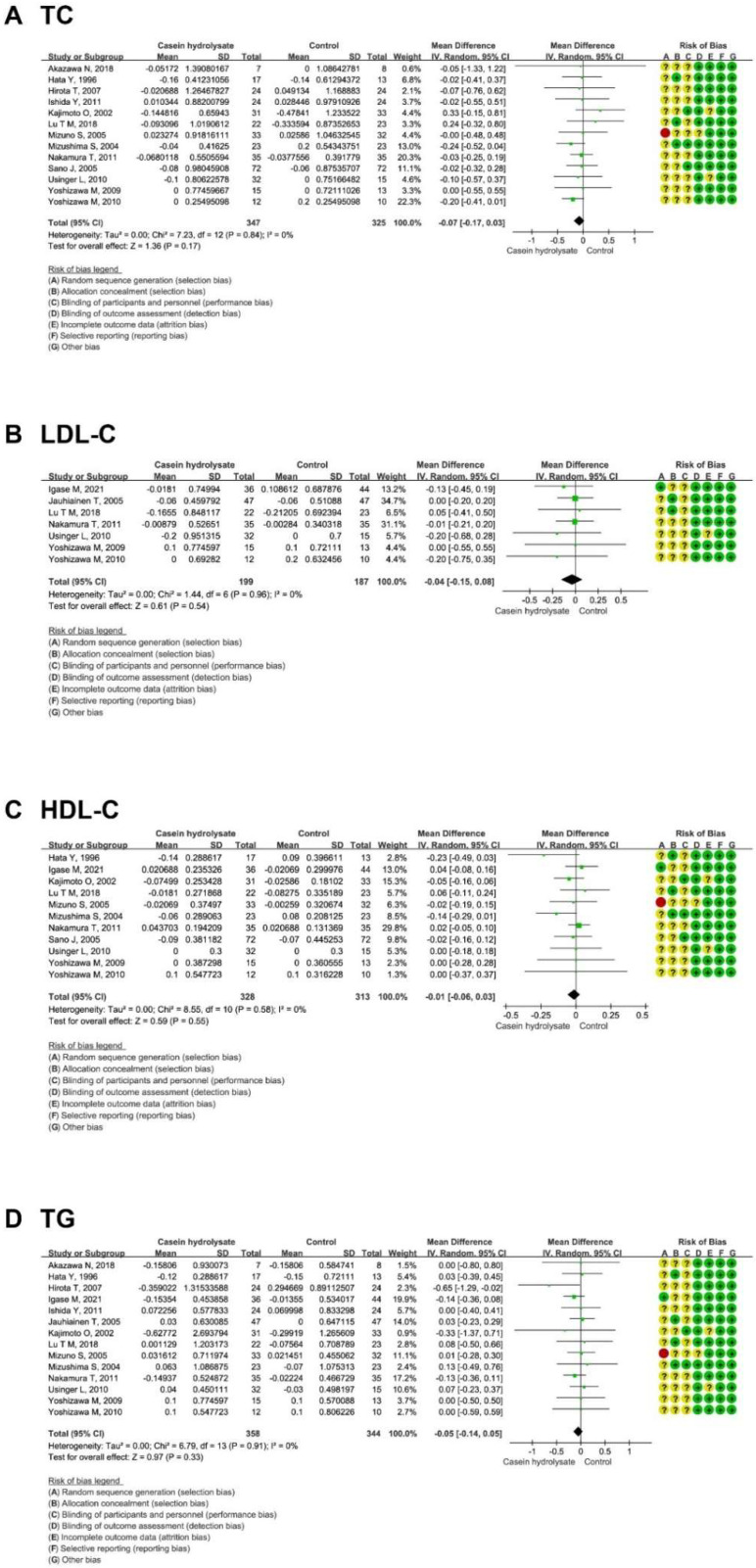
Effect of casein hydrolysate supplementation on blood lipids: (**A**) TG, (**B**) TC, (**C**) LDL-C, and (**D**) HDL-C. Each domain of risk of bias is represented by a circle: a green circle for low risk, a yellow circle for unclear risk, and a red circle for high risk.

Subsequently, the overall effects of casein hydrolysate on blood lipids (TC, LDL-C, HDL-C, and TG) were assessed. It was shown that casein hydrolysate had no significant effect on blood lipids: TC (−0.07 mmol/L; 95% CI: −0.17, 0.03 mmol/L; *p* = 0.17), LDL (−0.04 mmol/L; 95% CI: −0.15, 0.08 mmol/L; *p* = 0.54), HDL (−0.01 mmol/L; 95% CI: −0.06, 0.03 mmol/L; *p* = 0.55), and TG (−0.05 mmol/L; 95% CI: −0.14, 0.05 mmol/L; *p* = 0.33) (Figure 3). The funnel plot and Egger’s linear regression test of blood lipids showed that no publication bias existed (*P*_TC_ = 0.304; *P*_LDL_ = 0.483; *P*_HDL_ = 0.396; *P*_TG_ = 0.473) (Figure S1, Table S2). Sensitivity analysis identified no small-study effects and the results are reliable (Figure S2).

### 3.4. Effect of Casein Hydrolysate on FBG

**Figure 4:**
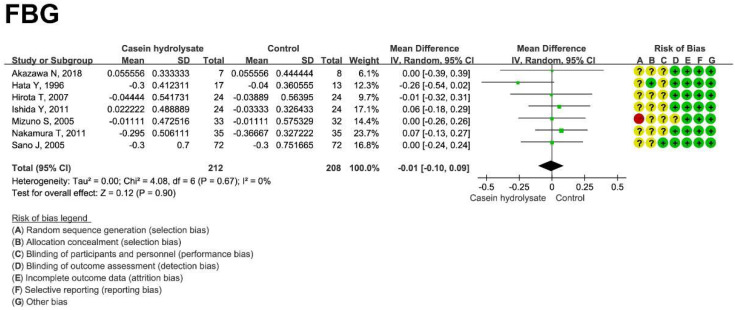
Effect of casein hydrolysate on FBG. Each domain of risk of bias is represented by a circle: a green circle for low risk, a yellow circle for unclear risk, and a red circle for high risk.

There was no effect of casein hydrolysate on FBG (−0.01 mmol/L; 95% CI: −0.10, 0.09 mmol/L; *p* = 0.90) compared with the control diets (Figure 4). No publication bias (*p* = 0.430) was observed (Figure S1, Table S2). The result of FBG was reliable, as revealed by sensitivity analysis (Figure S2).

### 3.5. Stratified Analysis of the Effects of Casein Hydrolysate on BP

**Figure 5:**
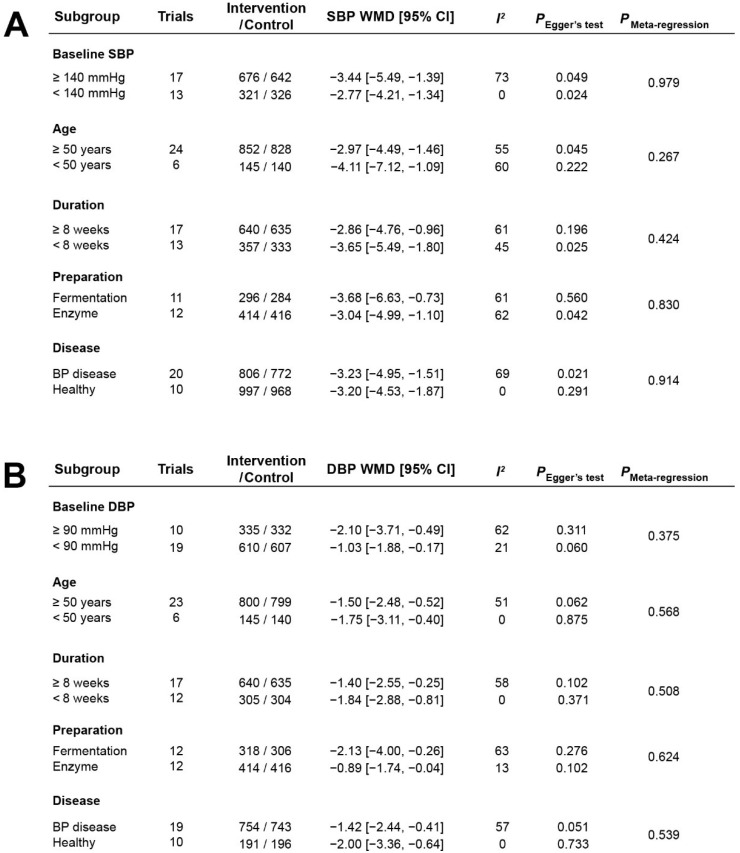
Effects of casein hydrolysate on SBP (**A**) and DBP (**B**) in stratified analyses.

To further identify the effect of casein hydrolysate on BP, we conducted a stratified analysis of 30 trials (Figure 5 and Figures S3–S12). Participants with elevated BP at baseline (SBP ≥ 140 mmHg; DBP ≥ 90 mmHg) showed no significant decreased effect in SBP (−3.44 mmHg; 95% CI: −5.49, −1.39 mmHg, *p* = 0.001) and DBP (−2.10 mmHg; 95% CI: −3.71, −0.49 mmHg; *p* = 0.01) compared to participants with normal BP (SBP: −2.77 mmHg; 95% CI: −4.21, −1.34 mmHg; *p* = 0.0002. DBP: −1.03 mmHg; 95% CI: −1.88, −0.17 mmHg; *p* = 0.02) (Figures S3 and S8). Younger participants (<50 years) had no statistically significant reductions in SBP (−4.11 mmHg; 95% CI: −7.12, −1.09 mmHg; *p* = 0.008) and DBP (−1.75 mmHg; 95% CI: −3.11, −0.40 mmHg; *p* = 0.01) compared to the older participants (SBP: −2.97 mmHg; 95% CI: −4.49, −1.46 mmHg; *p* = 0.0001. DBP: −1.50 mmHg; 95% CI: −2.48, −0.52 mmHg; *p* = 0.003) (Figures S4 and S9). Short-term casein hydrolysate intervention (<8 weeks) also had no effect on reducing both SBP (−3.65 mmHg; 95% CI: −5.49, −1.80 mmHg; *p* = 0.0001) and DBP (−1.84 mmHg; 95% CI: −2.88, −0.81 mmHg; *p* = 0.0005) compared with the long-term intervention (SBP: −2.86 mmHg; 95% CI: −4.76, −0.96 mmHg; *p* = 0.003. DBP: −1.40 mmHg; 95% CI: −2.55, −0.25 mmHg; *p* = 0.02) (Figures S5 and S10). Fermentation preparation of casein hydrolysate had no reduction effect on SBP (−3.68 mmHg; 95% CI: −6.63, −0.73 mmHg; *p* = 0.01) and DBP (−2.13 mmHg; 95% CI: −4.00, −0.26 mmHg; *p* = 0.03) compared with enzymatic preparation method (SBP: −3.04 mmHg; 95% CI: −4.99, −1.10 mmHg; *p* = 0.002. DBP: −0.89 mmHg; 95% CI: −1.74, −0.04 mmHg; *p* = 0.04) (Figures S6 and S11). In addition, the blood pressure reduction effect was not different between the healthy participants receiving casein hydrolysate (SBP: −3.18 mmHg; 95% CI: −5.10, −1.26 mmHg; *p* = 0.001. DBP: −2.00 mmHg; 95% CI: −3.36, −0.64 mmHg; *p* = 0.004) and the participants with blood pressure disorders (SBP: −3.23 mmHg; 95% CI: −4.95, −1.51 mmHg; *p* = 0.0002. DBP: −1.42 mmHg; 95% CI: −2.44, −0.41 mmHg; *p* = 0.006) (Figures S7 and S12). Taken together, no difference was observed in the BP-reducing effect of casein hydrolysate among participants stratified by baseline BP, age, duration of the intervention, preparation methods, and disease status.

### 3.6. Stratified Analysis of the Effects of Casein Hydrolysate on Blood Lipids and Blood Glucose

**Figure 6:**
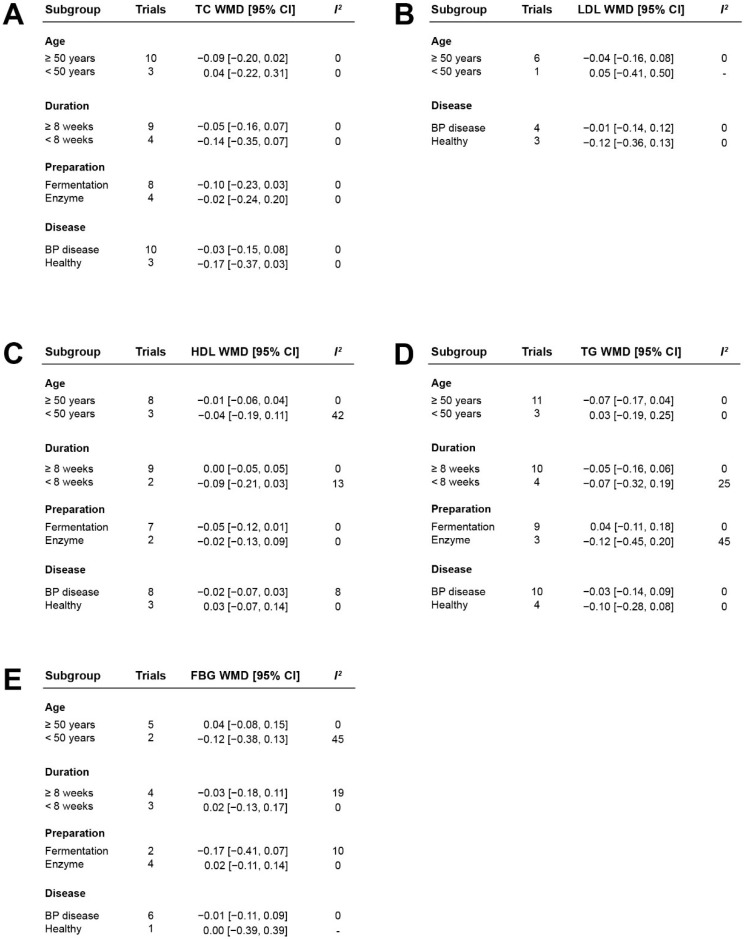
The effect of casein hydrolysate in stratified analyses of TC (**A**), LDL (**B**), HDL (**C**), TG (**D**), and FBG (**E**) compared with control diets.

To further assess the effect of casein hydrolysate on blood lipids and blood glucose, we conducted a stratified analysis for extracted data (Figure 6 and Figures S13–S30). We analyzed 13 trials to explore the effect of casein hydrolysate supplementation on TC. TC were not altered by casein hydrolysate supplementation in subgroups (Figure 6A). LDL levels were not changed in the subgroups according to the age or disease status of participants (Figure 6B). HDL levels from 11 trials were analyzed in subgroups according to similar risk factors, and no effect was observed in different subgroups (Figure 6C). Data extracted from 14 trials were used for the stratified analyses of TG, and casein hydrolysate supplementation had no reduction effect on TG according to the age of participants, duration of intervention, preparation method, or disease status (Figure 6D). Data from the seven relevant trials were used to explore the effect of casein hydrolysate on FBG in different subgroups, and casein hydrolysate had no effect on FBG concentrations in different subgroups (Figure 6E).

## 4. Discussion

To determine the association between casein hydrolysate consumption and different CVD risk factors, the current systematic review and meta-analyses evaluated the effects of casein hydrolysate on SBP, DBP, TC, LDL, HDL, and FBG. Twenty-six RCTs were included to investigate the effect of casein hydrolysate supplementation on multiple CVD risk factors. Casein hydrolysate significantly reduced SBP (−3.20 mmHg; 95% CI: −4.53, −1.87 mmHg; *p* < 0.00001) and DBP (−1.50 mmHg; 95% CI: −2.31, −0.69 mmHg; *p* = 0.0003) compared with control diets, but no effect on TC (−0.07 mmol/L; 95% CI: −0.17, 0.03 mmol/L; *p* = 0.17), LDL-C (−0.04 mmol/L; 95% CI: −0.15, 0.08 mmol/L; *p* = 0.54), HDL-C (−0.01 mmol/L; 95% CI: −0.06, 0.03 mmol/L; *p* = 0.55), TG (−0.05 mmol/L; 95% CI: −0.14, 0.05 mmol/L; *p* = 0.33), or FBG (−0.01 mmol/L; 95% CI: −0.10, 0.09 mmol/L; *p* = 0.90). Furthermore, no difference in reducing BP was observed among participants stratified by different baseline BP, age, duration of the intervention, preparation methods, and disease status. Consistent with the BP results of stratified analyses, no significant difference was observed in blood lipids and blood glucose among participants stratified by age, duration of the intervention, preparation methods, and disease status. To our knowledge, this is the first systematic review comprehensively assessing the overall effects of the available RCTs of casein hydrolysate supplementation on CVD risk factors. Our study possesses several strengths. First, our analysis reflected the most updated and comprehensive assessment of the effects of casein hydrolysate on multiple CVD risk factors. Second, we provided stratified analyses to identify potential covariates modifying the effect of casein hydrolysate supplementation on CVD risk factors. Third, a detailed sensitivity analysis and publication bias analysis were performed to ensure the robustness of the results. The findings of this study had several public health implications. For instance, our findings supported the consumption of casein hydrolysate in the population at risk for the prevention of CVDs.

The BP decreasing mechanism of casein hydrolysate is that it contains various bioactive peptides during fermentation or enzymatic preparation of casein, such as isoleucine-proline-proline and valine-proline-proline, the most studied casein hydrolysate [34,35]. These bioactive peptides were shown to have a vasodilative effect by inhibiting ACE activity in human interventional studies [36,37]. ACE is the critical enzyme converting angiotensin I into the vasoconstrictor angiotensin II, making vasodilator bradykinin inactivation and eliciting BP elevation [38]. In addition to the ACE inhibitory effect, these bioactive peptides in casein hydrolysate also inhibit the activity of renin and endothelin-converting enzyme, which interacts with bradykinin receptors and Ca^2+^ channels, modulating sympathetic nervous activity to reduce blood pressure [39,40]. Furthermore, bioactive peptides derived from casein hydrolysate have been reported to display preventive effects on cerebrovascular aging and neurovascular diseases [37,41]. However, the underlying mechanism still requires further investigation. Compared with casein hydrolysate, the CVD preventive effect of casein protein was less investigated [42,43,44]. Casein is regarded as a slow digesting protein in the stomach that delays the gastric emptying process [43,45]. Weight reduction and improving blood lipid have been reported in persons receiving casein protein intervention [44]. Therefore, the health impact of casein and casein hydrolysate may be different.

The potent ACE-inhibitory peptides from natural casein hydrolysate have been widely studied as a safe alternative to synthetic ACE inhibitors for hypertension treatment [15,46]. Different from synthetic ACE inhibitors (e.g., captopril, fosinopril, and ramipril), the administration of which is associated with unfavorable side effects, including persistent coughs, angioneurotic edema, skin rash, taste disturbance, and renal impairment [47,48]. Natural ACE-inhibitory peptides from casein hydrolysate could avoid these side effects. Production of casein-derived peptides is achieved by fermentation or enzymatic methods in commercial production. The fermentation process utilized lactic acid bacteria [49], while the enzymatic methods utilized pepsin and trypsin to release the active peptides [50]. These preparation methods possess adequate safety, high reliability, and quite effective advantages.

In animals receiving oral gavage of casein hydrolysate, no behavioral, organic, or histopathological differences were observed between intervention and control groups [51,52,53]. In interventional studies performed on humans, the lowest (3 mg/day) and highest dose (52.5 mg/day) of ACE-inhibitory peptides from casein hydrolysate displayed no adverse reactions [54,55]. In summary, the consumption of casein hydrolysate is relatively safe within a reasonable dose. Considering the antihypertensive effect of casein hydrolysate, casein hydrolysate may generate a synergistic effect with synthetic ACE inhibitors and reduce the amount of synthetic ACE inhibitor administration in hypertensive patients.

There are published systematic reviews and meta-analyses investigating the influences of casein hydrolysate supplementation on BP and other CVD risk factors [28,29,30,31,56,57]. Our findings suggest that casein hydrolysate supplementation is beneficial for blood pressure control, consistent with previous systematic reviews. However, limitations still existed in these studies. For instance, these studies did not include the most recent studies on CVD risk factors. A recent systematic review and meta-analysis about the effects of casein hydrolysate on blood pressure was published in 2015 [28]. An increasing number of new RCTs that met the inclusion criteria was published, potentially leading to different discoveries. Moreover, some published meta-analyses studies lack detailed subgroup analysis [29,31]. Turpeinen et al. [29] only conducted the overall analysis on SBP (−4 mmHg; 95% CI: −5.9, −2.1 mmHg; *p* < 0.001) and DBP (−1.9 mmHg; 95% CI: −3.1 to −0.8 mmHg; *p* < 0.001). In another study, subgroup analysis only included a small number of participants, which may lead to a biased conclusion [30]. Two studies only concentrated on the pooled effects of casein hydrolysate on BP in specific populations [56,57]. Cicero et al. [57] reported that the casein hydrolysate could moderately reduce SBP (−1.28 mmHg; 95% CI: −2.09, −0.48 mmHg; *p* = 0.0017) in European subjects. Compared with these published meta-analysis articles, our review conducted pooled and detailed subgroup analyses of casein-derived hydrolysate supplementation on multiple CVD risk factors. We also incorporated recent published RCTs investigating the effect of casein hydrolysate on BP (see Supplemental Material in Table S1). Although our updated results still support the BP lowering effect of casein hydrolysate, we also found that the BP reduction effect of casein hydrolysate was similar for participants stratified by baseline BP, age, duration of the intervention, preparation methods, and disease status. These results suggest that the antihypertension activities of casein hydrolysate could be generalized to diverse populations.

Reviewing the RCTs articles that met inclusion criteria, few articles investigated the effects of casein hydrolysate consumption on blood lipids or blood glucose in humans [58]. Jauhiainen et al. [58] reported that casein hydrolysate intake for three months showed no differences in blood lipids compared with the control group for mildly hypertensive subjects. Until now, there has been a lack of systematic review and meta-analysis on blood lipids and blood glucose of casein hydrolysate. Therefore, we extracted relevant blood lipids and blood glucose data from numerous BP RCTs to explore the effects of casein hydrolysate intake. Different from previous systematic reviews of casein hydrolysate, the influences of casein hydrolysate supplementation on blood lipids and blood glucose were also analyzed by the overall and stratified analyses in our study. Our results indicated that casein hydrolysate intervention did not affect blood lipids or blood glucose in both overall and stratified analyses. Although casein hydrolysate contains beneficial bioactive peptides with anti-inflammatory and antioxidant activities, which are expected to display improving effects on blood lipids and blood glucose, such effects may require a longer-term intervention of casein hydrolysate. In the current analyses, the intervention duration of most studies was less than 2 months. Therefore, a long-term intervention (e.g., 6 months or more) is needed to capture the influences of casein hydrolysate on blood lipids or blood glucose.

Limitations still exist in the present study. First, several results were obtained from a small number of RCTs, especially in some stratified analyses, which may lead to biased results. Second, although the risk of bias in all eligible studies was adequately represented in the figures, some included RCT studies did not report sufficient information on the random sequence generation and allocation concealment. Third, some results may have significant heterogeneity and publication bias, which potentially influences the reliability of the results. Fourth, the BP reduction in casein hydrolysate may be also attributed to weight loss [59,60]. Casein hydrolysate supplementation induces satiety hormone (i.e., glucagon-like peptide 1, GLP-1) release, thus reducing portion size and food intake over time [60]. In addition, office blood pressure cannot be considered a specific indicator for an independent antihypertensive mechanism. Thus, the review requires weight and other clinical, biochemical results. Fifth, CVD risk factors are not equivalent to CVD risk; the CVD preventative effect still requires long-term evaluations in the future.

In conclusion, our study supports the beneficial role of casein hydrolysate intake in improving blood pressure. Unlike casein protein, casein hydrolysate displayed no effect on blood lipids and fasting blood glucose. Large-scale and long-term studies are still warranted in the future to validate the effect of casein hydrolysate on CVD incidence and the findings obtained in this study. In addition, our findings of this systematic review and meta-analysis have certain public health implications, and casein hydrolysate combined with other food-effective nutrients may generate potential synergistic effects on improving human health.

## Data Availability

Not applicable.

## Supplementary Materials

The following supporting information can be downloaded at: https://www.mdpi.com/article/10.3390/nu14194207/s1. Table S1: Characteristics of the publications included in the meta-analysis. Table S2: P value of Egger’s test analyses on SBP, DBP, TC, LDL, HDL, TG, and FBG from included RCTs articles. Figure S1: Funnel plots on SBP, DBP, TC, LDL, HDL, TG, FBG in the overall effect analysis. Figure S2: Sensitivity analysis on SBP, DBP, TC, LDL, HDL, TG, FBG in the overall effect analysis. Figure S3: Forest plots (A), sensitivity analysis (B), and funnel plots (C) on SBP (mmHg) in baseline SBP analysis. Figure S4: Forest plots (A), sensitivity analysis (B), and funnel plots (C) on SBP (mmHg) in the age analysis. Figure S5: Forest plots (A), sensitivity analysis (B), and funnel plots (C) on SBP (mmHg) in the duration analysis. Figure S6: Forest plots (A), sensitivity analysis (B), and funnel plots (C) on SBP (mmHg) in the preparation analysis. Figure S7: Forest plots (A), sensitivity analysis (B), and funnel plots (C) on SBP (mmHg) in the disease analysis. Figure S8: Forest plots (A), sensitivity analyses (B), and funnel plots (C) on DBP (mmHg) in baseline DBP analysis. Figure S9: Forest plots (A), sensitivity analyses (B), and funnel plots (C) on DBP (mmHg) in the age analysis. Figure S10: Forest plots (A), sensitivity analyses (B), and funnel plots (C) on DBP (mmHg) in the duration analysis. Figure S11: Forest plots (A), sensitivity analyses (B), and funnel plots (C) on DBP (mmHg) in the preparation analysis. Figure S12: Forest plots (A), sensitivity analyses (B), and funnel plots (C) on DBP (mmHg) in the disease analysis. Figure S13: Forest plots (A), sensitivity analysis (B), and funnel plots (C) on TC (mmol/L) in the age analysis. Figure S14: Forest plots (A), sensitivity analysis (B), and funnel plots (C) on TC (mmol/L) in the duration analysis. Figure S15: Forest plots (A), sensitivity analysis (B), and funnel plots (C) on TC (mmol/L) in the preparation analysis. Figure S16: Forest plots (A), sensitivity analysis (B), and funnel plots (C) on TC (mmol/L) in the disease analysis. Figure S17: Forest plots (A), sensitivity analysis (B), and funnel plots (C) on LDL (mmol/L) in the age analysis. Figure S18: Forest plots (A), sensitivity analysis (B), and funnel plots (C) on LDL (mmol/L) in the disease analysis. Figure S19: Forest plots (A), sensitivity analysis (B), and funnel plots (C) on HDL (mmol/L) in the age analysis. Figure S20: Forest plots (A), sensitivity analysis (B), and funnel plots (C) on HDL (mmol/L) in the duration analysis. Figure S21: Forest plots (A), sensitivity analysis (B), and funnel plots (C) on HDL (mmol/L) in the preparation analysis. Figure S22: Forest plots (A), sensitivity analysis (B), and funnel plots (C) on HDL (mmol/L) in the disease analysis. Figure S23: Forest plots (A), sensitivity analysis (B), and funnel plots (C) on TG (mmol/L) in the age analysis. Figure S24: Forest plots (A), sensitivity analysis (B), and funnel plots (C) on TG (mmol/L) in the duration analysis. Figure S25: Forest plots (A), sensitivity analysis (B), and funnel plots (C) on TG (mmol/L) in the preparation analysis. Figure S26: Forest plots (A), sensitivity analysis (B), and funnel plots (C) on TG (mmol/L) in the disease analysis. Figure S27: Forest plots (A), sensitivity analysis (B), and funnel plots (C) on FBG (mmol/L) in the age analysis. Figure S28: Forest plots (A), sensitivity analysis (B), and funnel plots (C) on FBG (mmol/L) in the duration analysis. Figure S29: Forest plots (A), sensitivity analysis (B), and funnel plots (C) on FBG (mmol/L) in the preparation analysis. Figure S30: Forest plots (A), sensitivity analysis (B), and funnel plots (C) on FBG (mmol/L) in the disease analysis. References [61,62,63,64,65,66,67,68,69,70,71,72,73,74,75,76,77,78,79] are cited in the Supplementary Materials.
