# Supplementary material for: Effect of Casein Hydrolysate on Cardiovascular Risk Factors: A Systematic Review and Meta-Analysis of Randomized Controlled Trials
Source: Nutrients. 2022 Oct 9;14(19):4207. doi: 10.3390/nu14194207 (PMC9573574; doi:10.3390/nu14194207)
Supplement: Supplementary file 1 [file nutrients-14-04207-s001.zip › nutrients-1925071-supplementary.pdf]

Supplementary Materials

The search terms of casein hydrolysate were used to search:

("casein hydrolysate" [Supplementary Concept]) AND ("blood pressure"[Mesh])

("casein hydrolysate" [Supplementary Concept]) AND ("cholesterol" [Mesh])

("casein hydrolysate" [Supplementary Concept]) AND ("triglycerides" [Mesh])

("casein hydrolysate" [Supplementary Concept]) AND ("blood glucose" [Mesh])

(ALL=(casein hydrolysate)) AND ALL=(blood pressure)

(ALL=(casein hydrolysate)) AND ALL=(cholesterol)

(ALL=(casein hydrolysate)) AND ALL=(triglycerides)

(ALL=(casein hydrolysate)) AND ALL=(blood glucose)

The references of included RCTs in this article:

(1) Akazawa, N.; Hamasaki, A.; Tanahashi, K.; Kosaki, K.; Yoshikawa, T.; Myoenzono, K.; Maeda, S. Lactotripeptide Ingestion Increases Cerebral Blood Flow Velocity in Middle-Aged and Older Adults. Nutr. Res. N. Y. N 2018, 53, 61–66. https://doi.org/10.1016/j.nutres.2018.03.009.

(2) Cadée, J. A.; Chang, C.-Y.; Chen, C.-W.; Huang, C.-N.; Chen, S.-L.; Wang, C.-K. Bovine Casein Hydrolysate (C12 Peptide) Reduces Blood Pressure in Prehypertensive Subjects. Am. J. Hypertens. 2007, 20 (1), 1–5. https://doi.org/10.1016/j.amjhyper.2006.06.005.

(3) Cicero, A. F. G.; Rosticci, M.; Ferroni, A.; Bacchelli, S.; Veronesi, M.; Strocchi, E.; Borghi, C. Predictors of the Short-Term Effect of Isoleucine-Proline-Proline/Valine-Proline-Proline Lactotripeptides from Casein on Office and Ambulatory Blood Pressure in Subjects with Pharmacologically Untreated High-Normal Blood Pressure or First-Degree Hypertension. Clin. Exp. Hypertens. N. Y. N 1993 2012, 34 (8), 601–605. https://doi.org/10.3109/10641963.2012.681731.

(4) Engberink, M. F.; Schouten, E. G.; Kok, F. J.; van Mierlo, L. A. J.; Brouwer, I. A.; Geleijnse, J. M. Lactotripeptides Show No Effect on Human Blood Pressure: Results from a Double-Blind Randomized Controlled Trial. Hypertens. Dallas Tex 1979 2008, 51 (2), 399–405. https://doi.org/10.1161/HYPERTENSIONAHA.107.098988.

(5) Germino, F. W.; Neutel, J.; Nonaka, M.; Hendler, S. S. The Impact of Lactotripeptides on Blood Pressure Response in Stage 1 and Stage 2 Hypertensives. J. Clin. Hypertens. Greenwich Conn 2010, 12 (3), 153–159. https://doi.org/10.1111/j.1751-7176.2009.00250.x.

(6) Igase, M.; Okada, Y.; Igase, K.; Matsumoto, S.; Senzaki, K.; Ochi, M.; Ohyagi, Y.; Yamagishi, S.-I. Casein Hydrolysate Containing Milk-Derived Peptides Reduces Facial Pigmentation Partly by Decreasing Advanced Glycation End Products in the Skin: A Randomized Double-Blind Placebo-Controlled Trial. Rejuvenation Res. 2021, 24 (2), 97–103. https://doi.org/10.1089/rej.2020.2343.

(7) Ishida, Y.; Shibata, Y.; Fukuhara, I.; Yano, Y.; Takehara, I.; Kaneko, K. Effect of an Excess Intake of Casein Hydrolysate Containing Val-Pro-Pro and Ile-Pro-Pro in Subjects with Normal Blood Pressure, High-Normal Blood Pressure, or Mild Hypertension. Biosci. Biotechnol. Biochem. 2011, 75 (3), 427–433. https://doi.org/10.1271/bbb.100560.

(8) Jauhiainen, T.; Niittynen, L.; Orešič, M.; Järvenpää, S.; Hiltunen, T. P.; Rönnback, M.; Vapaatalo, H.; Korpela, R. Effects of Long-Term Intake of Lacto-tripeptides on Cardiovascular Risk Factors in Hypertensive Subjects. Eur. J. Clin. Nutr. 2012, 66 (7), 843–849. https://doi.org/10.1038/ejcn.2012.44.

(9) Mizuno, S.; Matsuura, K.; Gotou, T.; Nishimura, S.; Kajimoto, O.; Yabune, M.; Kajimoto, Y.; Yamamoto, N. Antihypertensive Effect of Casein Hydrolysate in a Placebo-Controlled Study in Subjects with High-Normal Blood Pressure and Mild Hypertension. Br. J. Nutr. 2005, 94 (1), 84–91. https://doi.org/10.1079/bjn20051422.

(10) Nakamura, T.; Mizutani, J.; Ohki, K.; Yamada, K.; Yamamoto, N.; Takeshi, M.; Takazawa, K. Casein Hydrolysate Containing Val-Pro-Pro and Ile-Pro-Pro Improves Central Blood Pressure and Arterial Stiffness in Hypertensive Subjects: A Randomized, Double-Blind, Placebo-Controlled Trial. Atherosclerosis 2011, 219 (1), 298–303. https://doi.org/10.1016/j.atherosclerosis.2011.06.007.

(11) Sano, J.; Ohki, K.; Higuchi, T.; Aihara, K.; Mizuno, S.; Kajimoto, O.; Nakagawa, S.; Kajimoto, Y.; Nakamura, Y. Effect of Casein Hydrolysate, Prepared with Protease Derived from Aspergillus Oryzae, on Subjects with High-Normal Blood Pressure or Mild Hypertension. J. Med. Food 2005, 8 (4), 423–430. https://doi.org/10.1089/jmf.2005.8.423.

(12) Usinger, L.; Jensen, L. T.; Flambard, B.; Linneberg, A.; Ibsen, H. The Antihypertensive Effect of Fermented Milk in Individuals with Prehypertension or Borderline Hypertension. J. Hum. Hypertens. 2010, 24 (10), 678–683. https://doi.org/10.1038/jhh.2010.4.

(13) van der Zander, K.; Bots, M. L.; Bak, A. A. A.; Koning, M. M. G.; de Leeuw, P. W. Enzymatically Hydrolyzed Lactotripeptides Do Not Lower Blood Pressure in Mildly Hypertensive Subjects. Am. J. Clin. Nutr. 2008, 88 (6), 1697–1702. https://doi.org/10.3945/ajcn.2008.26003.

(14) Yoshizawa, M.; Maeda, S.; Miyaki, A.; Misono, M.; Choi, Y.; Shimojo, N.; Ajisaka, R.; Tanaka, H. Additive Beneficial Effects of Lactotripeptides and Aerobic Exercise on Arterial Compliance in Postmenopausal Women. Am. J. Physiol. Heart Circ. Physiol. 2009, 297 (5), H1899-1903. https://doi.org/10.1152/ajpheart.00433.2009.

(15) Yoshizawa, M.; Maeda, S.; Miyaki, A.; Misono, M.; Choi, Y.; Shimojo, N.; Ajisaka, R.; Tanaka, H. Additive Beneficial Effects of Lactotripeptides Intake with Regular Exercise on Endothelium-Dependent Dilatation in Postmenopausal Women. Am. J. Hypertens. 2010, 23 (4), 368–372. https://doi.org/10.1038/ajh.2009.270.

(16) Kajimoto, O.; Kurosaki, T.; Mizutani, J.; Ikeda, N.; Kaneko, K.; Aihara, K.; Yabune, M.; Nakamura, Y. Antihypertensive Effects of Liquid Yogurts Containing "Lactotripeptides (VPP, IPP)" in Mild Hypertensive Subjects. J Nutr Food 2002, 5.

(17) Hata, Y.; Yamamoto, M.; Ohni, M.; Nakajima, K.; Nakamura, Y.; Takano, T. A Placebo-Controlled Study of the Effect of Sour Milk on Blood Pressure in Hypertensive Subjects. Am. J. Clin. Nutr. 1996, 64 (5), 767–771. https://doi.org/10.1093/ajcn/64.5.767.

(18) Jauhiainen, T.; Vapaatalo, H.; Poussa, T.; Kyrönpalo, S.; Rasmussen, M.; Korpela, R. Lactobacillus Helveticus Fermented Milk Lowers Blood Pressure in Hypertensive Subjects in 24-h Ambulatory Blood Pressure Measurement. Am. J. Hypertens. 2005, 18 (12 Pt 1), 1600–1605. https://doi.org/10.1016/j.amjhyper.2005.06.006.

(19) Jauhiainen, T.; Rönnback, M.; Vapaatalo, H.; Wuolle, K.; Kautiainen, H.; Groop, P.-H.; Korpela, R. Long-Term Intervention with Lactobacillus Helveticus Fermented Milk Reduces Augmentation Index in Hypertensive Subjects. Eur. J. Clin. Nutr. 2010, 64 (4), 424–431. https://doi.org/10.1038/ejcn.2010.3.

(20) Mizushima, S.; Ohshige, K.; Watanabe, J.; Kimura, M.; Kadowaki, T.; Nakamura, Y.; Tochikubo, O.; Ueshima, H. Randomized Controlled Trial of Sour Milk on Blood Pressure in Borderline Hypertensive Men. Am. J. Hypertens. 2004, 17 (8), 701–706. https://doi.org/10.1016/j.amjhyper.2004.03.674.

(21) Seppo, L.; Jauhiainen, T.; Poussa, T.; Korpela, R. A Fermented Milk High in Bioactive Peptides Has a Blood Pressure-Lowering Effect in Hypertensive Subjects. Am. J. Clin. Nutr. 2003, 77 (2), 326–330. https://doi.org/10.1093/ajcn/77.2.326.

(22) Lu, T.; Chiu, H.; Lu, Y.; Han, Y.-C.; Shen, Y.; Kamesh, V.; Golovinskaia, O.; Wang, C.-K. Efficacy of Fermented Goat Milk on Blood Pressure in Prehypertensive Adults: A Randomized, Placebo-controlled, Clinical Trial. J. Food Biochem. 2017, 42. https://doi.org/10.1111/jfbc.12474.

(23) Cicero, A. F. G.; Colletti, A.; Rosticci, M.; Cagnati, M.; Urso, R.; Giovannini, M.; Borghi, C.; D'Addato, S. Effect of Lactotripeptides (Isoleucine-Proline-Proline/Valine-Proline-Proline) on Blood Pressure and Arterial Stiffness Changes in Subjects with Suboptimal Blood Pressure Control and Metabolic Syndrome: A Double-Blind, Randomized, Crossover Clinical Trial. Metab. Syndr. Relat. Disord. 2016, 14 (3), 161–166. https://doi.org/10.1089/met.2015.0093.

(24) Cicero, A. F. G.; Rosticci, M.; Veronesi, M.; Bacchelli, S.; Strocchi, E.; Melegari, C.; Grandi, E.; Borghi, C. Hemodynamic Effects of Lactotripeptides from Casein Hydrolysate in Mediterranean Normotensive Subjects and Patients with High-Normal Blood Pressure: A Randomized, Double-Blind, Crossover Clinical Trial. J. Med. Food 2010, 13 (6), 1363–1368. https://doi.org/10.1089/jmf.2009.0253.

(25) Hirota, T.; Ohki, K.; Kawagishi, R.; Kajimoto, Y.; Mizuno, S.; Nakamura, Y.; Kitakaze, M. Casein Hydrolysate Containing the Antihypertensive Tripeptides Val-Pro-Pro and Ile-Pro-Pro Improves Vascular Endothelial Function Independent of Blood Pressure-Lowering Effects: Contribution of the Inhibitory Action of Angiotensin-Converting Enzyme. Hypertens. Res. Off. J. Jpn. Soc. Hypertens. 2007, 30 (6), 489–496. https://doi.org/10.1291/hypres.30.489.

(26) Yamasue, K.; Morikawa, N.; Mizushima, S.; Tochikubo, O. The Blood Pressure Lowering Effect of Lactotripeptides and Salt Intake in 24-h Ambulatory Blood Pressure Measurements. Clin. Exp. Hypertens. N. Y. N 1993 2010, 32 (4), 214–220. https://doi.org/10.3109/10641963.2010.491885.

Table S1. Characteristics of the publications included in the meta-analysis.

Article	Study design	Source of hydroly-sate	Preparation method	Intervention diet	Control diet	Extracted out-come(s)	Duration	Health status	Population size n (Ctrl, Inter)	Location	Mean age, y
Akazawa N, 2018	R, DB	Milk	Fermentation	LTPs	Control	SBP,DBP,TG,TC,FBG	8 W	1	15 (8,7)	Japan	52-75
Cadée, 2007	R, DB	Casein	-	C12 peptide	Control	SBP,DBP	4 W	3	48 (24,24)	China	>50
Cicero A. F. G, 2012	R, DB	Casein	-	LTPs	Control	SBP,DBP	4 W	2,4	164 (82,82)	Italy	43.85±11.11
Cicero A. F. G, 2010	R, SB, C	Casein	Enzyme	LTPs	Control	SBP,DBP	4 W	1,2	55 (55,55)	Italy	40.3±9.8
Cicero A. F. G, 2016	R, DB, C	Casein	Enzyme	LTPs	Control	SBP,DBP	4 W	7	40 (40,40)	Italy	40-75
Engberink M. F, 2008	R, DB	Casein	Fermentation	LTPs	Control	SBP,DBP	8 W	2,6	67 (32,35)	Dutch	58.8±9.1
Engberink M. F, 2008	R, DB	Casein	Enzyme	LTPs	Control	SBP,DBP	8 W	2,6	64 (32,32)	Dutch	54.2±8.8
Engberink M. F, 2008	R, DB	Casein	Synthesis	LTPs	Control	SBP,DBP	8 W	2,6	68 (32,36)	Dutch	59.5±8.2
Germino F W, 2010	R, DB	-	-	LTPs	Control	SBP	6 W	4,5	81 (29,52)	America	32–78
Hirota T, 2007	R, DB, C	Casein	Enzyme	LTPs	Control	SBP,DBP,TG,TC, FBG	1 W	4	24 (24,24)	Japan	54.1±8.1
Igase M, 2021	R, DB	Casein	-	LTPs	Control	SBP,DBP,TG,LDL,HDL	48 W	1	80 (44,36)	Japan	71.7±9.2
Ishida Y, 2011	R, DB	Casein	Enzyme	LTPs	Control	SBP,DBP,TG,TC, FBG	4 W	1,2,4	48 (24,24)	Japan	49.3±11.9
Jauhiainen T, 2012	R, DB	-	-	Peptide	Control	SBP,DBP	3 M	4	89 (44,45)	Finland	49±5
Mizuno S, 2005	R, SB	Casein	Enzyme	IPP, VPP	Control	SBP,DBP,TG,TC,HDL, FBG	6 W	4	41 (20, 21)	Japan	46±14.7
Mizuno S, 2005	R, SB	Casein	Enzyme	IPP, VPP	Control	SBP,DBP,TG,TC,HDL, FBG	6 W	2	24 (12, 12)	Japan	42.6±12.1
Nakamura T, 2011	R, DB	-	-	LTPs	Control	SBP,DBP,TG,TC,LDL,HDL, FBG	8 W	6	90 (45,45)	Japan	50-69

Sano J, 2005	R, DB	Casein	Enzyme	LTPs	Control	SBP,DBP,TC,HDL, FBG	12 W	2,4	144 (72,72)	Japan	51±10
Usinger L, 2010	R, DB	-	Fermentation	LTPs	Control	TG,TC,LDL,HDL	8 W	3,6	48 (15,32)	Denmark	54±11
Van D, 2008	R, DB	Casein	Enzyme	ELTP	Control	SBP,DBP	8 W	4	271(137,134)	Dutch	60±7
Yamasue K, 2010	R, DB, C	-	Fermentation	LTPs	Control	SBP,DBP	8 W	2	22 (22,22)	Japan	63.3±4.6
Yoshizawa M, 2010	R	-	Fermentation	LTPs	Control	SBP,DBP,TG,TC,LDL,HDL	8 W	1	22 (10,12)	Japan	58±1
Yoshizawa M, 2009	R	Casein	Fermentation	LTPs	Control	SBP,DBP,TG,TC,LDL,HDL	8 W	1	28 (13,15)	Japan	59±1
Kajimoto O, 2002	R, DB	Milk	Fermentation	LTPs	Control	SBP,DBP,TG,TC, HDL	8 W	4	64 (33,31)	Japan	50±11
Hata Y, 1996	R	Milk	Fermentation	Sour milk	Control	SBP,DBP,TG,TC,HDL, FBG	8 W	6	30 (13,17)	Japan	40-86
Jauhiainen T, 2005	R, DB	Milk	Fermentation	Sour milk	Control	SBP,DBP,TG,LDL	10 W	6	94 (47,47)	Finland	55±11
Jauhiainen T, 2010	R, DB	Milk	Fermentation	Peptide	Control	SBP,DBP	24 W	6	89 (45,44)	Finland	49±5
Mizushima S, 2004	R, DB	Milk	Fermentation	Sour milk	Control	SBP,DBP,TG,TC,HDL	4 W	6	46 (23,23)	Japan	48.5±8.9
Seppo L, 2002	R, DB	Milk	Fermentation	LTPs	Control	SBP,DBP	21 W	6	36 (17,19)	Finland	47.9±6.9
Lu T M， 2018	R, DB	Milk	Fermentation	LTPs	Control	TG,TC,LDL,HDL	8 W	3	45 (23,22)	China	30-65

Data are presented as mean ± sd or as a range.
Study design: R, randomized; DB, double-blinded; SB, single-blinded; C, crossover design.
Intervention diet: LTPs, lactotripeptides; IPP, Isoleucine-Proline-Proline; VPP, Valine-Proline-Proline.
Extracted outcome(s): SBP, systolic blood pressure; DBP, diastolic blood pressure; TG, triglyceride; TC, total cholesterol; HDL-C, high density lipoprotein cholesterol; LDL-C, low density lipoprotein-cholesterol; FBG, fasting blood pressure.
Duration: W, week; M, month.
Health status: 1, health; 2, high-normal blood pressure; 3, prehypertension; 4, Stage I hypertension (mild hypertension); 5, Stage II hypertension (moderate hypertension); 6, hypertension; 7, metabolic syndrome.
Population size: Ctrl, control group; Inter, intervention group.
-, not reported.

Table S2. P value of Egger's test analyses on SBP, DBP, TC, LDL, HDL, TG, and FBG from included RCTs articles.

	$P_{\text{Egger's test}}$
SBP	0.01
DBP	0.03
TC	0.304
LDL	0.483
HDL	0.396
TG	0.473
FBG	0.430

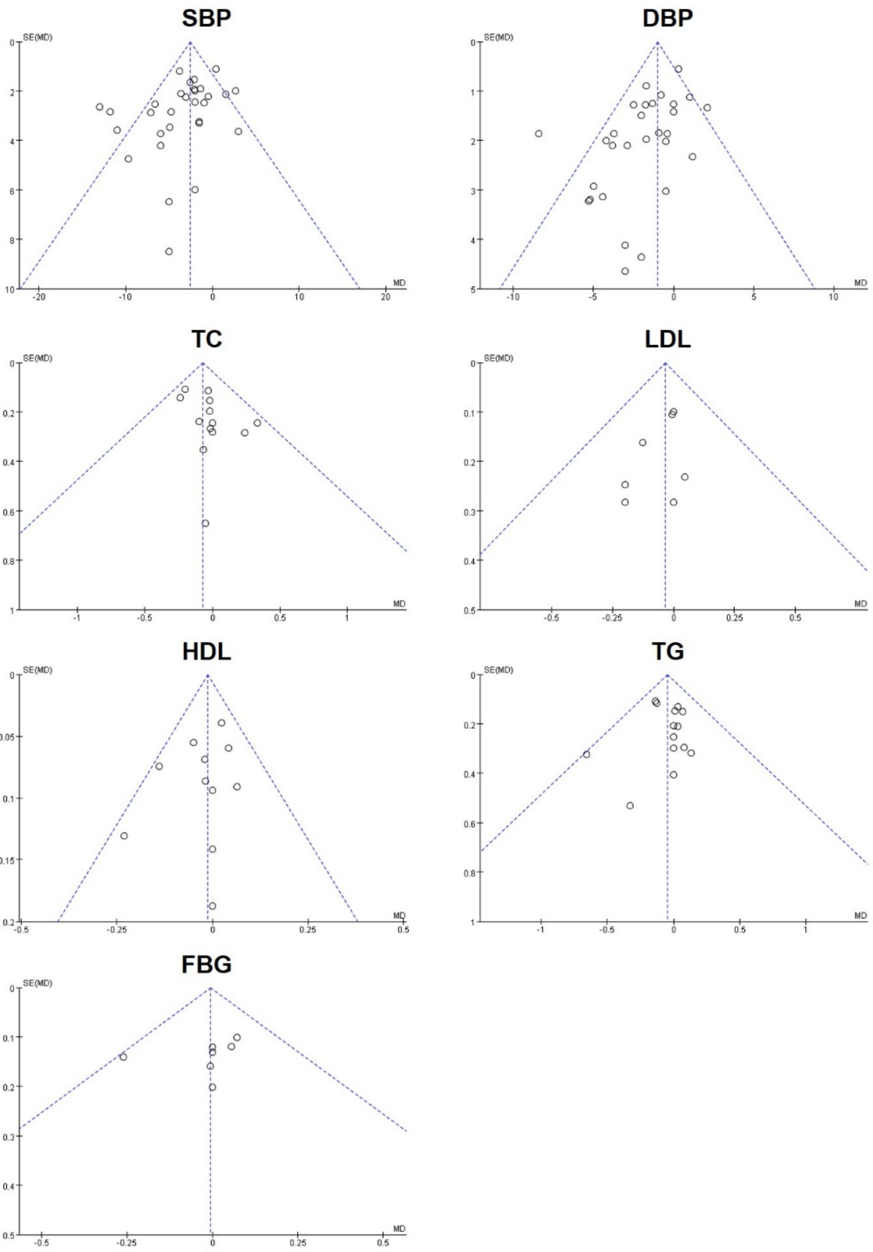

Figure S1. Funnel plots on SBP, DBP, TC, LDL, HDL, TG, FBG in the overall effect analysis.

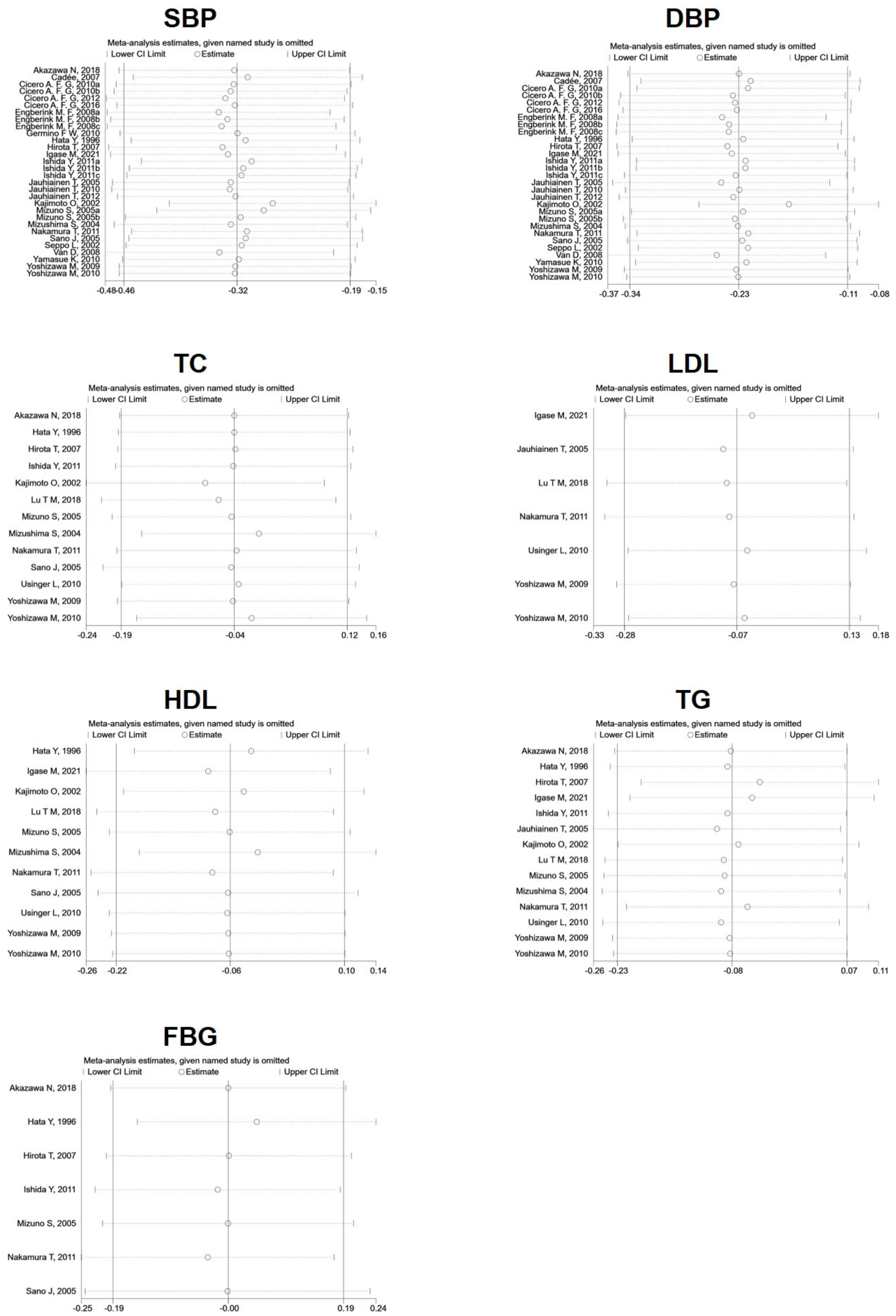

Figure S2. Sensitivity analysis on SBP, DBP, TC, LDL, HDL, TG, FBG in the overall effect analysis.

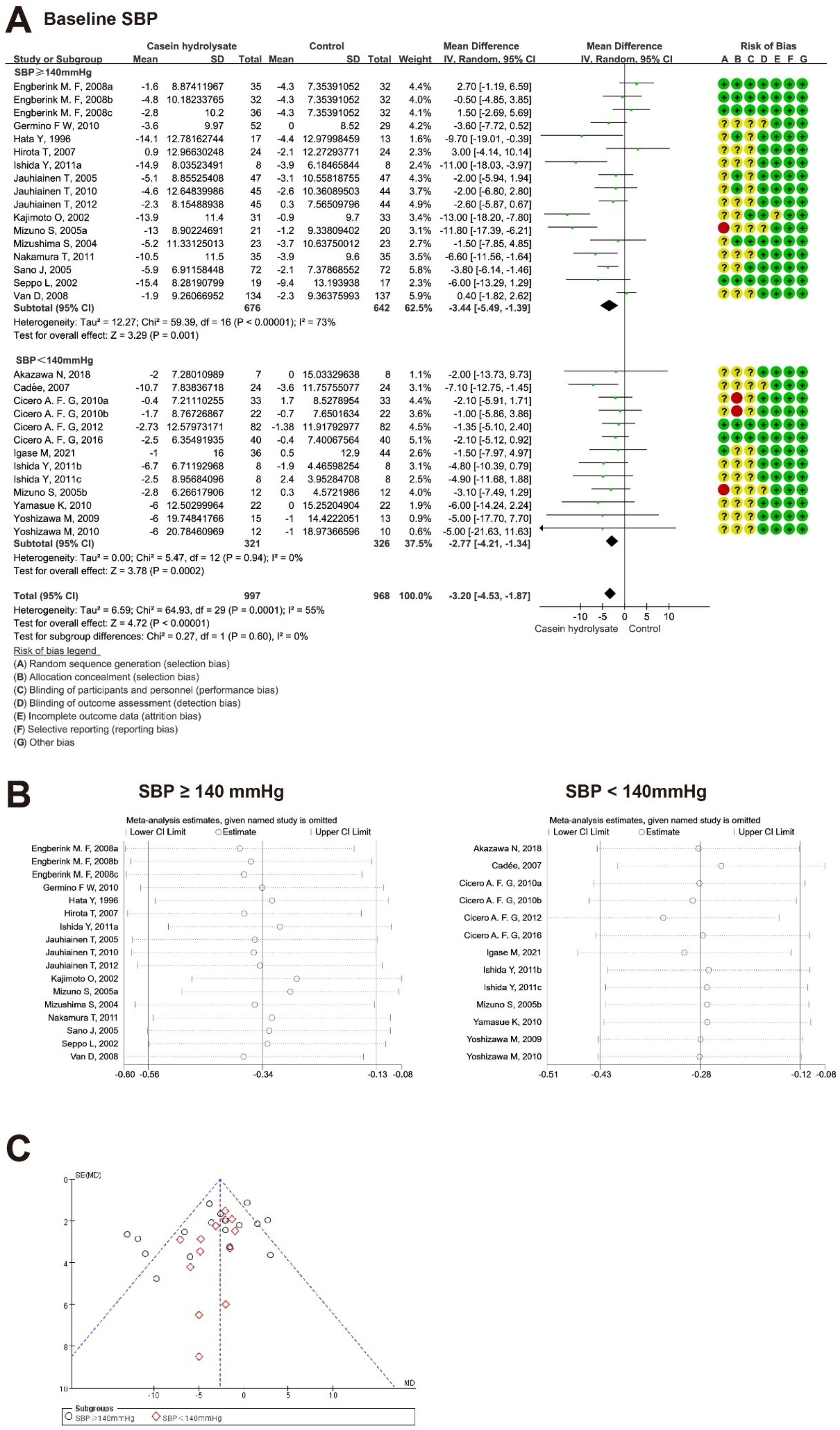

Figure S3. Forest plots (A), sensitivity analysis (B), and funnel plots (C) on SBP (mmHg) in baseline SBP analysis. The results represented the changes of SBP in baseline SBP subgroup with the casein hydrolysate intervention in comparison with the control diet.

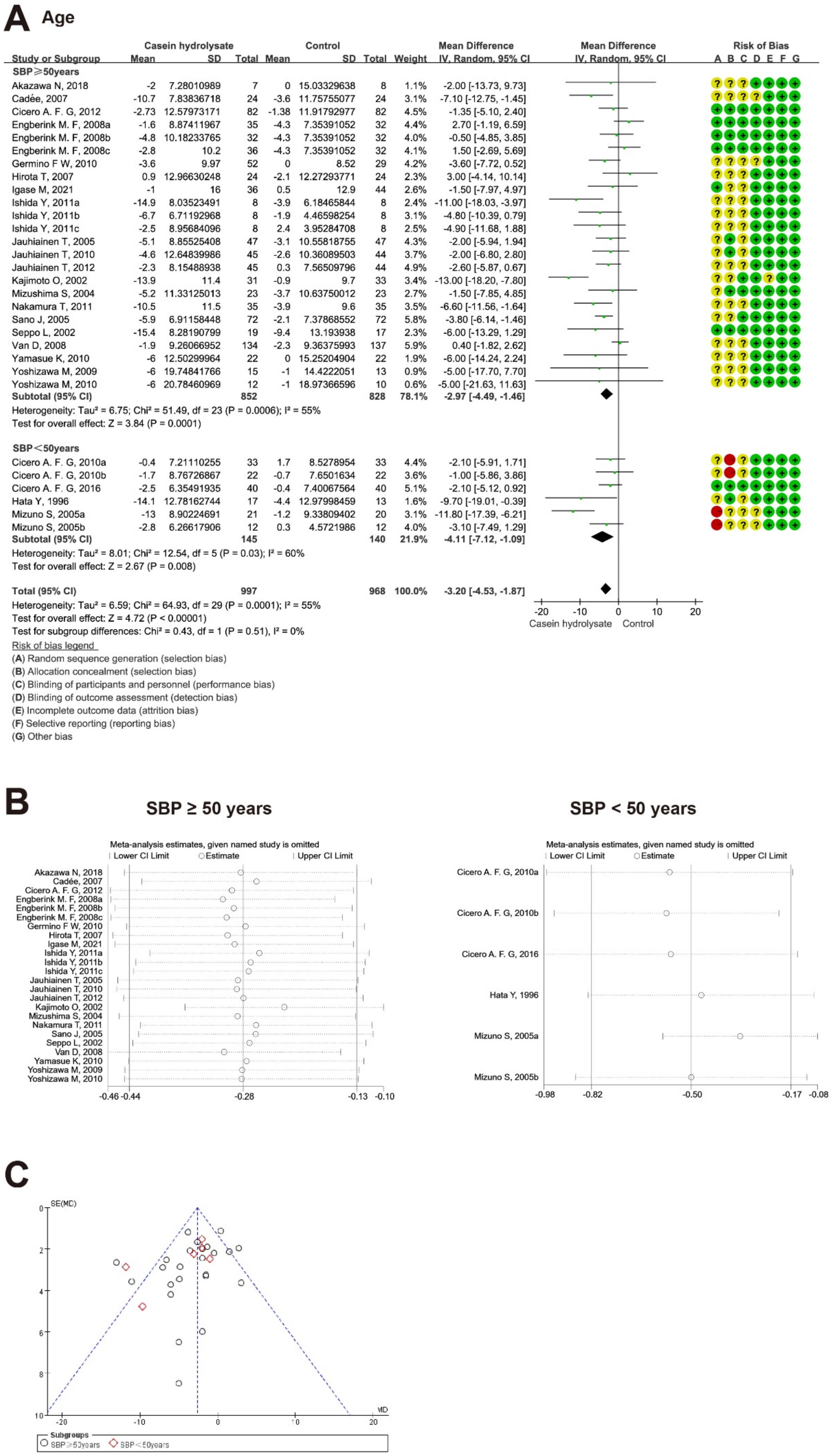

Figure S4. Forest plots (A), sensitivity analysis (B), and funnel plots (C) on SBP (mmHg) in the age analysis. The results represented the changes of SBP in the age subgroup with the casein hydrolysate intervention in comparison with the control diet.

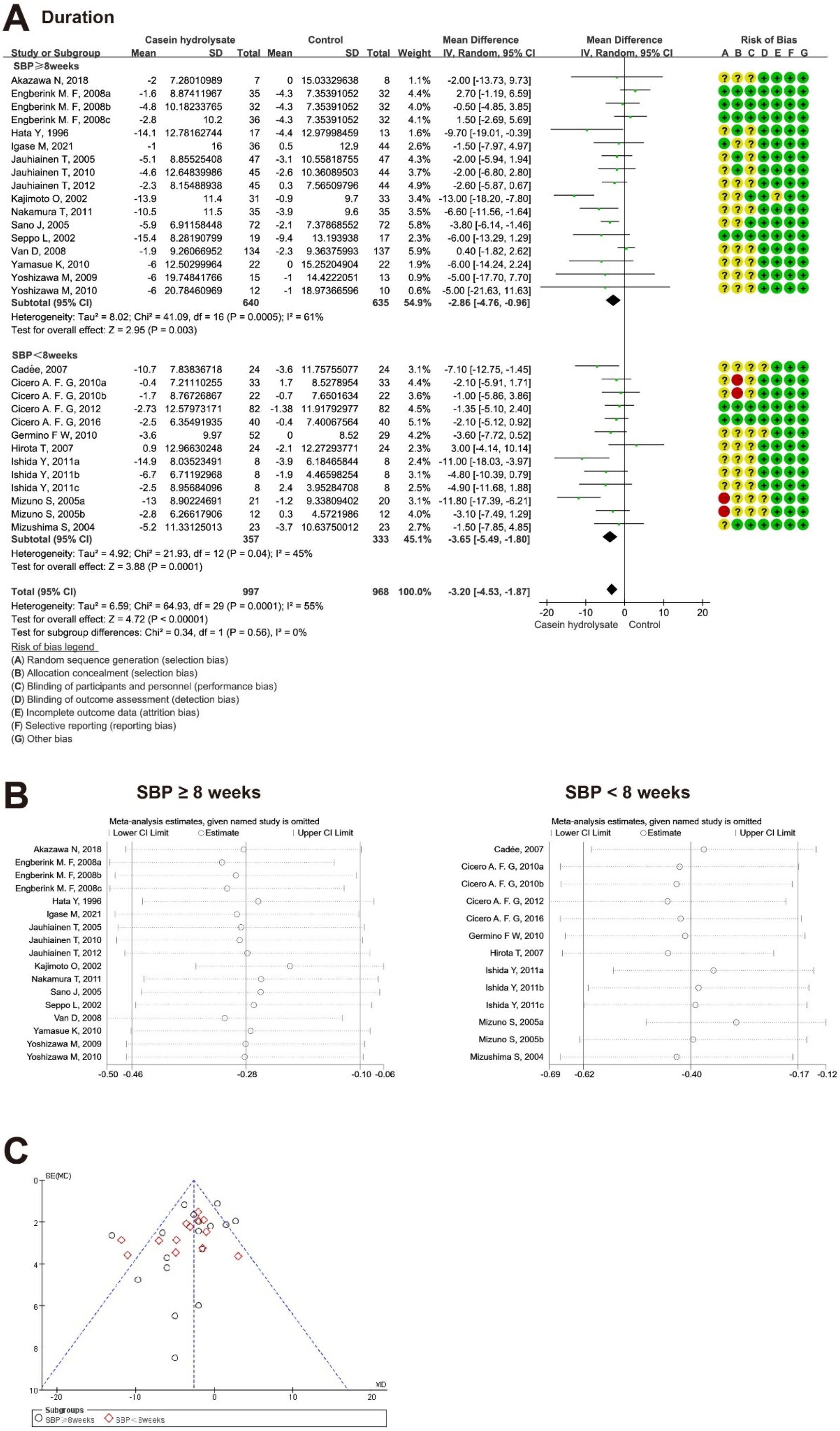

Figure S5. Forest plots (A), sensitivity analysis (B), and funnel plots (C) on SBP (mmHg) in the duration analysis. The results represented the changes of SBP in the duration subgroup with the casein hydrolysate intervention in comparison with the control diet.

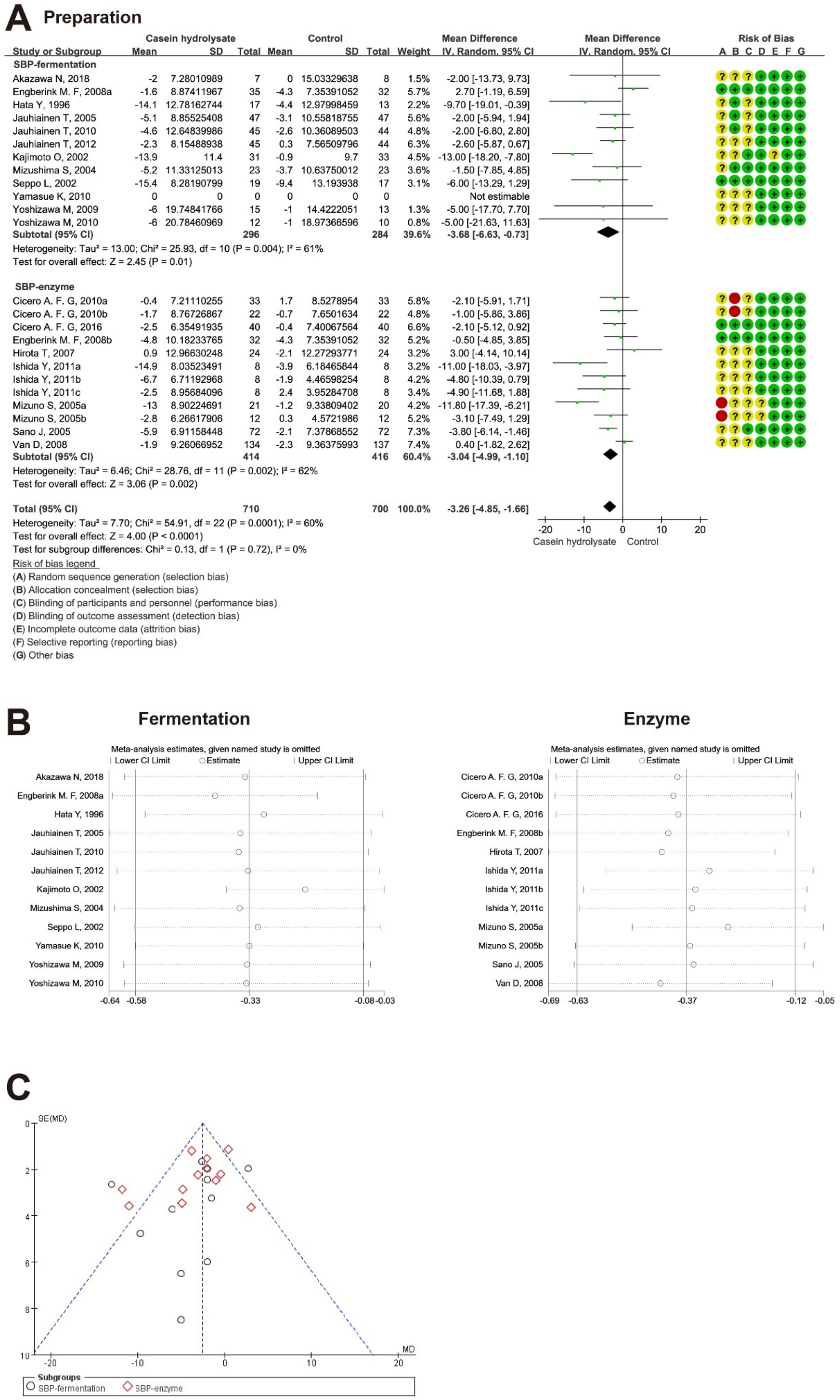

Figure S6. Forest plots (A), sensitivity analysis (B), and funnel plots (C) on SBP (mmHg) in the preparation analysis. The results represented the changes of SBP in the preparation subgroup with the casein hydrolysate intervention in comparison with the control diet.

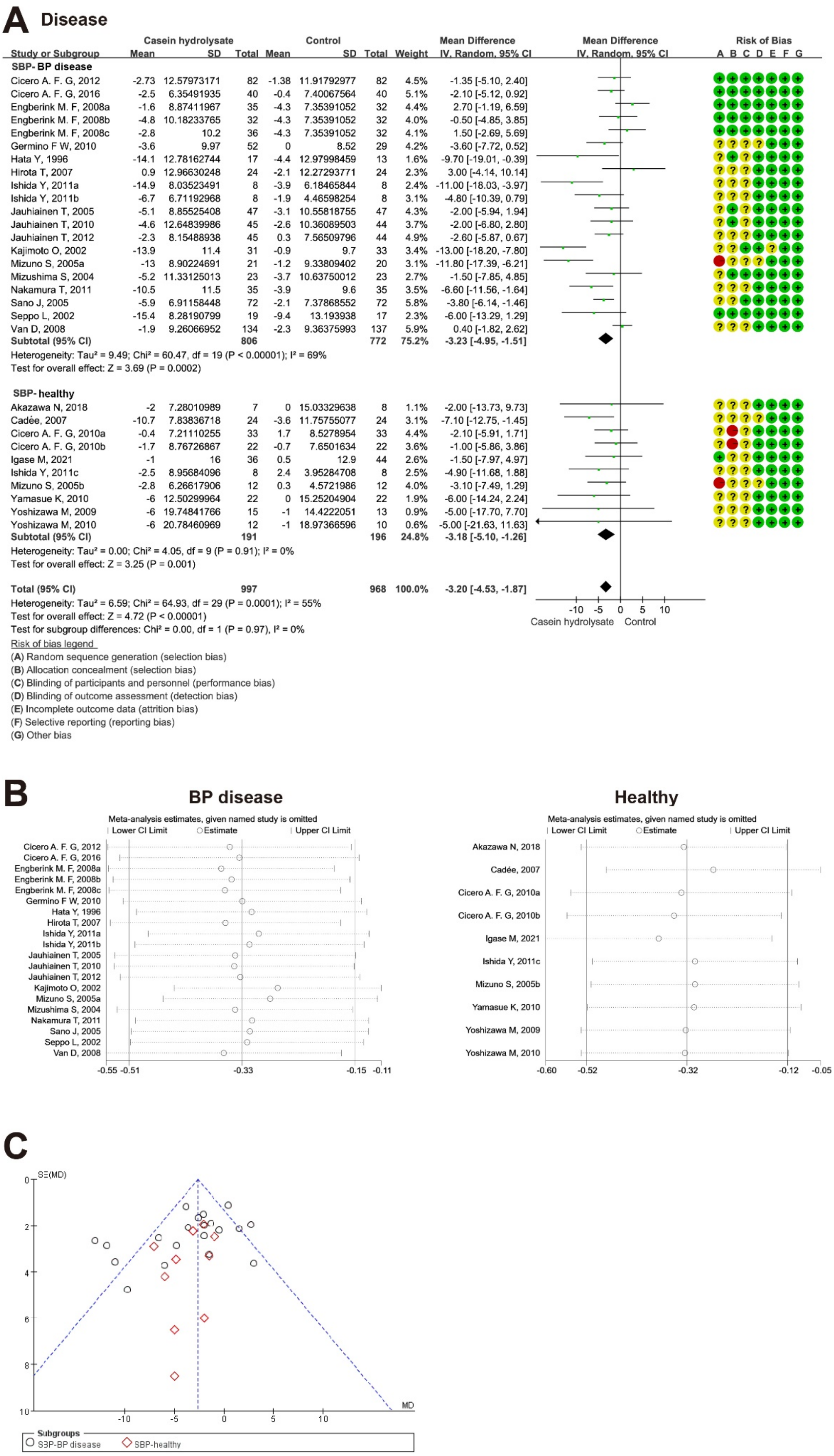
A Disease
Casein hydrolysate
Control
Mean Difference
Mean Difference
Risk of Bias
Study or Subgroup Mean SD Total Mean SD Total Weight IV, Random, 95% CI IV, Random, 95% CI A B C D E F G
SBP- BP disease
Cicero A. F. G, 2012 -2.73 12.57973171 82 -1.38 11.91792977 82 4.5% -1.35 [-5.10, 2.40]
Cicero A. F. G, 2016 -2.5 6.35491935 40 -0.4 7.40067564 40 5.1% -2.10 [-5.12, 0.92]
Engberink M. F, 2008a -1.6 8.87411967 35 -4.3 7.35391052 32 4.4% 2.70 [-1.19, 6.59]
Engberink M. F, 2008b -4.8 10.18233765 32 -4.3 7.35391052 32 4.0% -0.50 [-4.85, 3.85]
Engberink M. F, 2008c -2.8 10.2 36 -4.3 7.35391052 32 4.1% 1.50 [-2.69, 5.69]
Germino F W, 2010 -3.6 9.97 52 0 8.52 29 4.2% -3.60 [-7.72, 0.52]
Hata Y, 1996 -14.1 12.78162744 17 -4.4 12.97998459 13 1.6% -9.70 [-19.01, -0.39]
Hirota T, 2007 0.9 12.96630248 24 -2.1 12.27293771 24 2.3% 3.00 [-4.14, 10.14]
Ishida Y, 2011a -14.9 8.03523491 8 -3.9 6.18465844 8 2.4% -11.00 [-18.03, -3.97]
Ishida Y, 2011b -6.7 6.71192968 8 -1.9 4.46598254 8 3.1% -4.80 [-10.39, 0.79]
Jauhiainen T, 2005 -5.1 8.85525408 47 -3.1 10.55818755 47 4.3% -2.00 [-5.94, 1.94]
Jauhiainen T, 2010 -4.6 12.64839986 45 -2.6 10.36089503 44 3.7% -2.00 [-6.80, 2.80]
Jauhiainen T, 2012 -2.3 8.15488938 45 0.3 7.56509796 44 4.9% -2.60 [-5.87, 0.67]
Kajimoto O, 2002 -13.9 11.4 31 -0.9 9.7 33 3.4% -13.00 [-18.20, -7.80]
Mizuno S, 2005a -13 8.90224691 21 -1.2 9.33809402 20 3.1% -11.80 [-17.39, -6.21]
Mizushima S, 2004 -5.2 11.33125013 23 -3.7 10.63750012 23 2.7% -1.50 [-7.85, 4.85]
Nakamura T, 2011 -10.5 11.5 35 -3.9 9.6 35 3.5% -6.60 [-11.56, -1.64]
Sano J, 2005 -5.9 6.91158448 72 -2.1 7.37868552 72 5.8% -3.80 [-6.14, -1.46]
Seppo L, 2002 -15.4 8.28190799 19 -9.4 13.193938 17 2.3% -6.00 [-13.29, 1.29]
Van D, 2008 -1.9 9.26066952 134 -2.3 9.36375993 137 5.9% 0.40 [-1.82, 2.62]
Subtotal (95% CI) 806 772 75.2% -3.23 [-4.95, -1.51]
Heterogeneity: Tau² = 9.49; Chi² = 60.47, df = 19 (P < 0.00001); I² = 69%
Test for overall effect: Z = 3.69 (P = 0.0002)
SBP- healthy
Akazawa N, 2018 -2 7.28010989 7 0 15.03329638 8 1.1% -2.00 [-13.73, 9.73]
Cadée, 2007 -10.7 7.83836718 24 -3.6 11.75755077 24 3.1% -7.10 [-12.75, -1.45]
Cicero A. F. G, 2010a -0.4 7.21110255 33 1.7 8.5278954 33 4.4% -2.10 [-5.91, 1.71]
Cicero A. F. G, 2010b -1.7 8.76726867 22 -0.7 7.6501634 22 3.6% -1.00 [-5.86, 3.86]
Igase M, 2021 -1 16 36 0.5 12.9 44 2.6% -1.50 [-7.97, 4.97]
Ishida Y, 2011c -2.5 8.95684096 8 2.4 3.95284708 8 2.5% -4.90 [-11.68, 1.88]
Mizuno S, 2005b -2.8 6.26617906 12 0.3 4.5721986 12 4.0% -3.10 [-7.49, 1.29]
Yamasue K, 2010 -6 12.50299964 22 0 15.25204904 22 1.9% -6.00 [-14.24, 2.24]
Yoshizawa M, 2009 -6 19.74841766 15 -1 14.4222051 13 0.9% -5.00 [-17.70, 7.70]
Yoshizawa M, 2010 -6 20.78460969 12 -1 18.97366596 10 0.6% -5.00 [-21.63, 11.63]
Subtotal (95% CI) 191 196 24.8% -3.18 [-5.10, -1.26]
Heterogeneity: Tau² = 0.00; Chi² = 4.05, df = 9 (P = 0.91); I² = 0%
Test for overall effect: Z = 3.25 (P = 0.001)
Total (95% CI) 997 968 100.0% -3.20 [-4.53, -1.87]
Heterogeneity: Tau² = 6.59; Chi² = 64.93, df = 29 (P = 0.0001); I² = 55%
Test for overall effect: Z = 4.72 (P < 0.00001)
Test for subgroup differences: Chi² = 0.00, df = 1 (P = 0.97), I² = 0%
-10 -5 0 5 10
Casein hydrolysate Control
Risk of bias legend
(A) Random sequence generation (selection bias)
(B) Allocation concealment (selection bias)
(C) Blinding of participants and personnel (performance bias)
(D) Blinding of outcome assessment (detection bias)
(E) Incomplete outcome data (attrition bias)
(F) Selective reporting (reporting bias)
(G) Other bias
B
BP disease
Meta-analysis estimates, given named study is omitted
Lower CI Limit Estimate Upper CI Limit
Cicero A. F. G, 2012
Cicero A. F. G, 2016
Engberink M. F, 2008a
Engberink M. F, 2008b
Engberink M. F, 2008c
Germino F W, 2010
Hata Y, 1996
Hirota T, 2007
Ishida Y, 2011a
Ishida Y, 2011b
Jauhiainen T, 2005
Jauhiainen T, 2010
Jauhiainen T, 2012
Kajimoto O, 2002
Mizuno S, 2005a
Mizushima S, 2004
Nakamura T, 2011
Sano J, 2005
Seppo L, 2002
Van D, 2008
-0.55 -0.51 -0.33 -0.15 -0.11
Healthy
Meta-analysis estimates, given named study is omitted
Lower CI Limit Estimate Upper CI Limit
Akazawa N, 2018
Cadée, 2007
Cicero A. F. G, 2010a
Cicero A. F. G, 2010b
Igase M, 2021
Ishida Y, 2011c
Mizuno S, 2005b
Yamasue K, 2010
Yoshizawa M, 2009
Yoshizawa M, 2010
-0.60 -0.52 -0.32 -0.12 -0.05
C
SE(MD)
0 2 4 6 8 10
MD
-10 -5 0 5 10
Subgroups
SBP-BP disease
SBP-healthy

Figure S7. Forest plots (A), sensitivity analysis (B), and funnel plots (C) on SBP (mmHg) in the disease analysis. The results represented the changes of SBP in the disease subgroup with the casein hydrolysate intervention in comparison with the control diet.

A **Baseline DBP**

Study or Subgroup	Casein hydrolysate Mean	SD	Total	Control Mean	SD	Total	Weight	Mean Difference IV, Random, 95% CI
**DBP≥90mmHg**								
Hirota T, 2007	0.3	8.21735967	24	-0.9	7.92275205	24	2.4%	1.20 [-3.37, 5.77]
Ishida Y, 2011a	-7.1	6.45600496	8	-1.8	6.4501938	8	1.4%	-5.30 [-11.62, 1.02]
Jauhiainen T, 2005	-1.1	5.27909378	47	-2.1	5.61968047	47	5.7%	1.00 [-1.20, 3.20]
Jauhiainen T, 2010	-3.7	7.98846307	45	-1.7	6.05207836	44	4.3%	-2.00 [-4.94, 0.94]
Jauhiainen T, 2012	-1.6	4.99278942	45	-0.8	5.26267684	44	5.8%	-0.80 [-2.93, 1.33]
Kajimoto O, 2002	-9.1	7.5	31	-0.7	7.4	33	3.3%	-8.40 [-12.05, -4.75]
Mizuno S, 2005a	-4.2	7.61183289	21	-1.3	5.78705452	20	2.8%	-2.90 [-7.03, 1.23]
Mizushima S, 2004	-2	4.50937505	23	-0.3	8.32500009	23	3.0%	-1.70 [-5.57, 2.17]
Sano J, 2005	-3.2	5.05618433	72	-1.5	5.65022124	72	6.7%	-1.70 [-3.45, 0.05]
Seppo L, 2002	-9.3	4.79478884	19	-5.5	7.42159013	17	2.7%	-3.80 [-7.93, 0.33]
**Subtotal (95% CI)**			**335**			**332**	**38.0%**	**-2.10 [-3.71, -0.49]**
Heterogeneity: Tau² = 3.75; Chi² = 23.50, df = 9 (P = 0.005); I² = 62%								
Test for overall effect: Z = 2.56 (P = 0.01)								
**DBP<90mmHg**								
Akazawa N, 2018	-1	5.52268051	7	2	10.04987562	8	0.9%	-3.00 [-11.08, 5.08]
Cadée, 2007	-6.9	5.87877538	24	-2.7	7.83836718	24	3.0%	-4.20 [-8.12, -0.28]
Cicero A. F. G, 2010a	-1.3	6.06671245	33	1.2	4.1575233	33	5.0%	-2.50 [-5.01, 0.01]
Cicero A. F. G, 2010b	-1.2	7.00285656	22	-0.7	6.35177141	22	2.9%	-0.50 [-4.45, 3.45]
Cicero A. F. G, 2012	-2.35	8.32164046	82	-0.6	8.07902531	82	5.0%	-1.75 [-4.26, 0.76]
Cicero A. F. G, 2016	-1.2	5.85021367	40	0.1	5.40092585	40	5.1%	-1.30 [-3.77, 1.17]
Engberink M. F, 2008a	-0.3	5.91607978	35	-2.4	5.09116882	32	4.8%	2.10 [-0.54, 4.74]
Engberink M. F, 2008b	-2.4	5.09116882	32	-2.4	5.09116882	32	5.1%	0.00 [-2.49, 2.49]
Engberink M. F, 2008c	-2.4	6.6	36	-2.4	5.09116882	32	4.5%	0.00 [-2.79, 2.79]
Hata Y, 1996	-6.6	10.30776406	17	-2.2	6.85054742	13	1.5%	-4.40 [-10.55, 1.75]
Igase M, 2021	1.9	7.9	36	2.8	8.6	44	3.3%	-0.90 [-4.52, 2.72]
Ishida Y, 2011b	-4.1	6.13514466	8	1.1	6.6370174	8	1.4%	-5.20 [-11.46, 1.06]
Ishida Y, 2011c	-0.1	6.83117852	8	0.4	5.1623638	8	1.6%	-0.50 [-6.43, 5.43]
Mizuno S, 2005b	-1.4	5.38934133	12	-1	3.59026461	12	3.2%	-0.40 [-4.06, 3.26]
Nakamura T, 2011	-4.7	8.7	35	-1	6.7	35	3.3%	-3.70 [-7.34, -0.06]
Van D, 2008	-0.8	4.63033476	134	-1.1	4.68187996	137	8.2%	0.30 [-0.81, 1.41]
Yamasue K, 2010	-4	10.25597387	22	1	9.10878697	22	1.7%	-5.00 [-10.73, 0.73]
Yoshizawa M, 2009	-4	9.87420883	15	-2	12.74754878	13	0.8%	-2.00 [-10.54, 6.54]
Yoshizawa M, 2010	-2	10.39230485	12	1	11.18033989	10	0.7%	-3.00 [-12.09, 6.09]
**Subtotal (95% CI)**			**610**			**607**	**62.0%**	**-1.03 [-1.88, -0.17]**
Heterogeneity: Tau² = 0.67; Chi² = 22.78, df = 18 (P = 0.20); I² = 21%								
Test for overall effect: Z = 2.35 (P = 0.02)								
**Total (95% CI)**			**945**			**939**	**100.0%**	**-1.50 [-2.31, -0.69]**

Heterogeneity: Tau² = 1.75; Chi² = 48.97, df = 28 (P = 0.008); I² = 43%
Test for overall effect: Z = 3.63 (P = 0.0003)
Test for subgroup differences: Chi² = 1.34, df = 1 (P = 0.25), I² = 25.1%

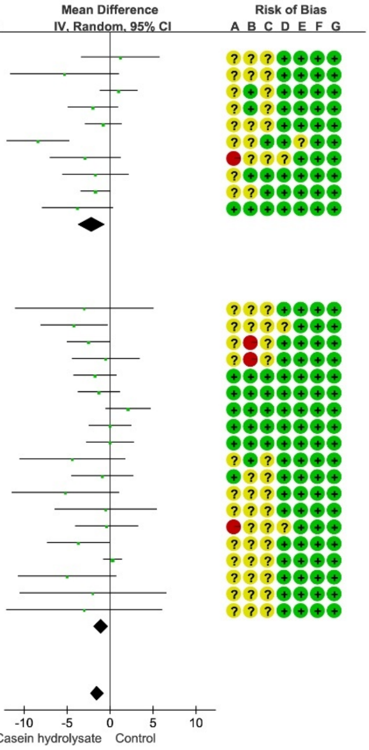

Risk of bias legend
(**A**) Random sequence generation (selection bias)
(**B**) Allocation concealment (selection bias)
(**C**) Blinding of participants and personnel (performance bias)
(**D**) Blinding of outcome assessment (detection bias)
(**E**) Incomplete outcome data (attrition bias)
(**F**) Selective reporting (reporting bias)
(**G**) Other bias

B

DBP ≥ 90 mmHg

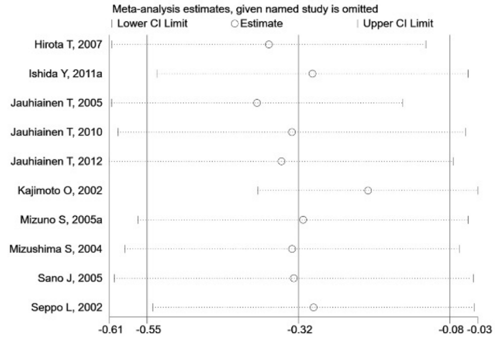

DBP < 90 mmHg

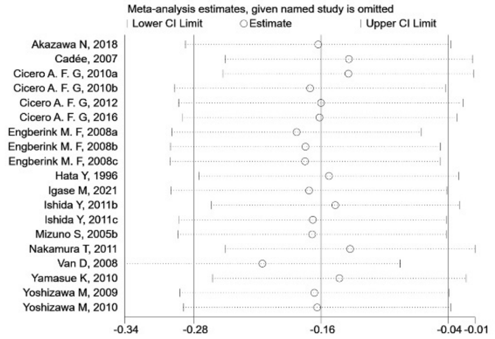

C

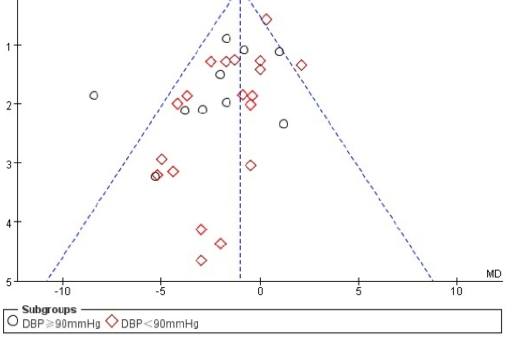

Figure S8. Forest plots (A), sensitivity analyses (B), and funnel plots (C) on DBP (mmHg) in baseline DBP analysis. The results represented the changes of DBP in baseline DBP subgroup with the casein hydrolysate intervention in comparison with the control diet.

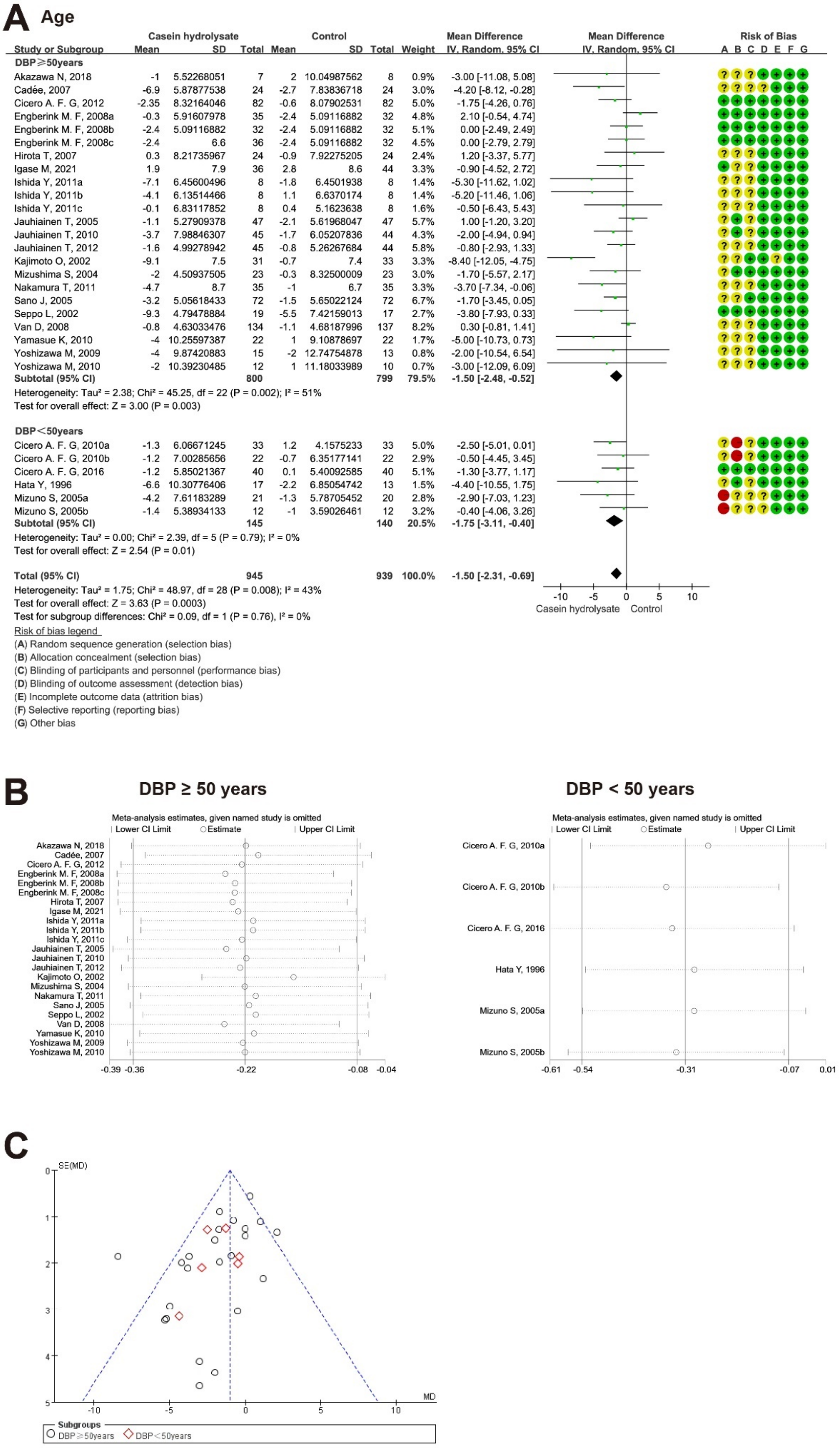

Figure S9. Forest plots (A), sensitivity analyses (B), and funnel plots (C) on DBP (mmHg) in the age analysis. The results represented the changes of DBP in the age subgroup with the casein hydrolysate intervention in comparison with the control diet.

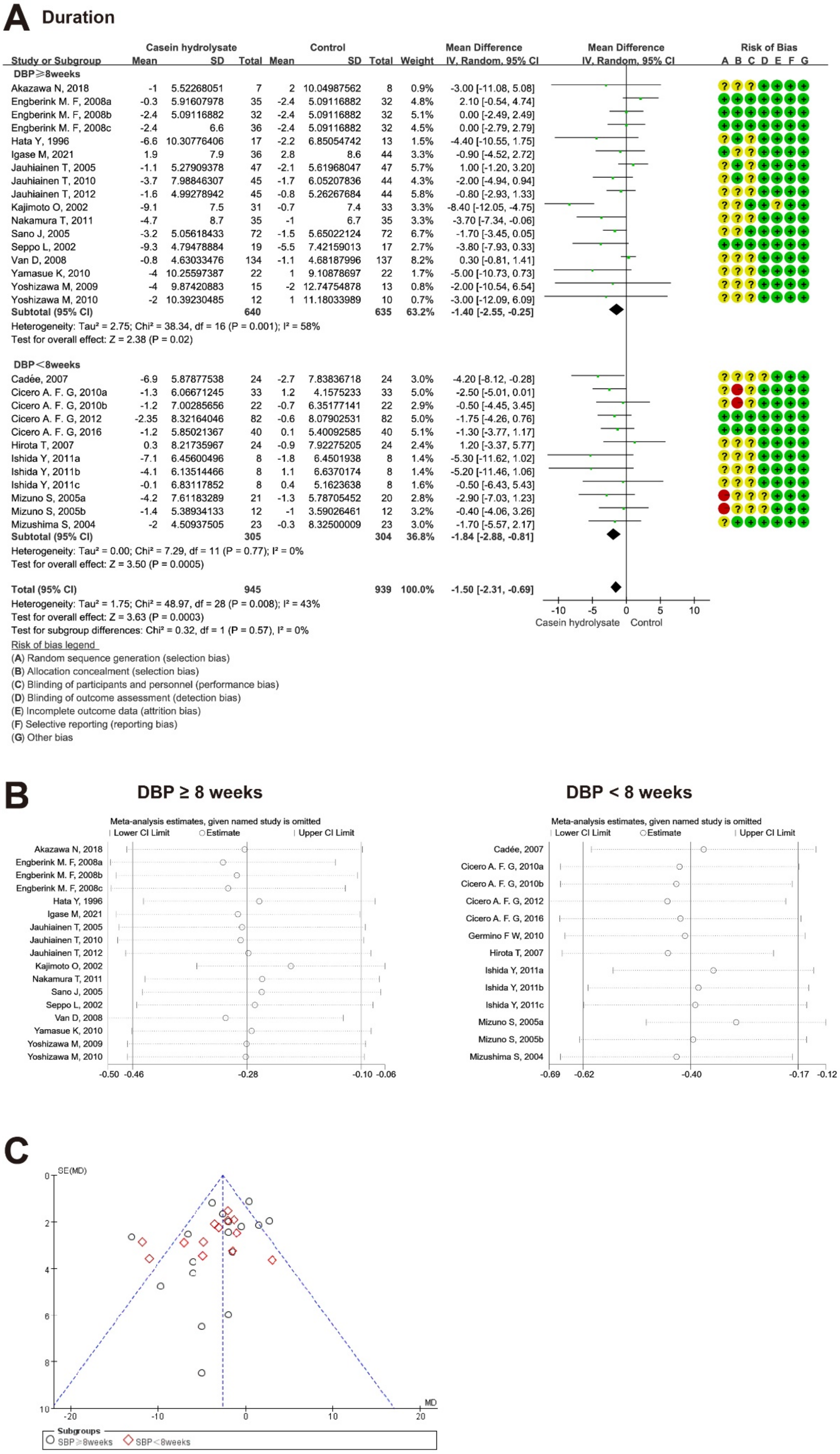

Figure S10. Forest plots (A), sensitivity analyses (B), and funnel plots (C) on DBP (mmHg) in the duration analysis. The results represented the changes of DBP in the duration subgroup with the casein hydrolysate intervention in comparison with the control diet.

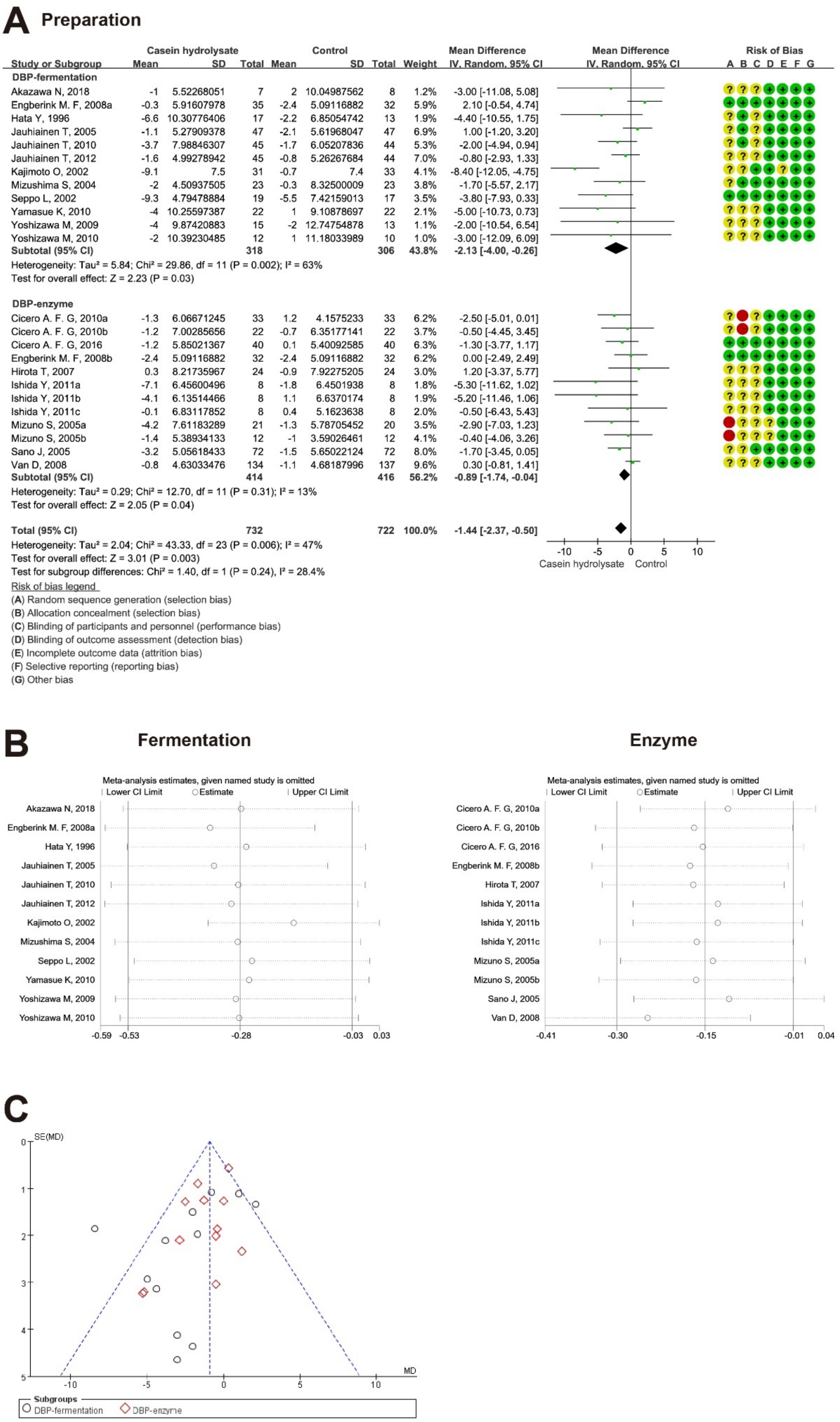

Figure S11. Forest plots (A), sensitivity analyses (B), and funnel plots (C) on DBP (mmHg) in the preparation analysis. The results represented the changes of DBP in the preparation subgroup with the casein hydrolysate intervention in comparison with the control diet.

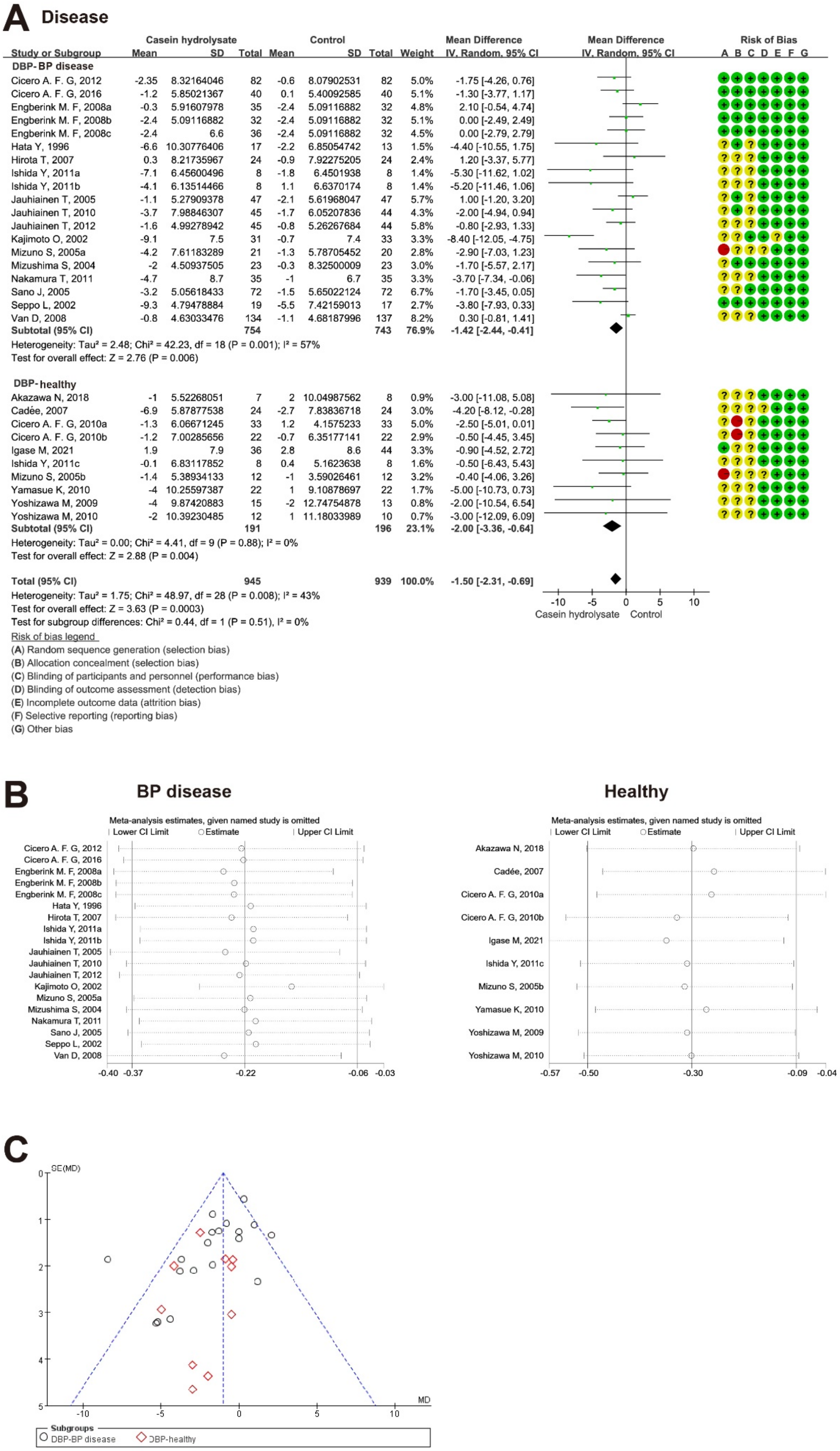

Figure S12. Forest plots (A), sensitivity analyses (B), and funnel plots (C) on DBP (mmHg) in the disease analysis. The results represented the changes of DBP in the disease subgroup with the casein hydrolysate intervention in comparison with the control diet.

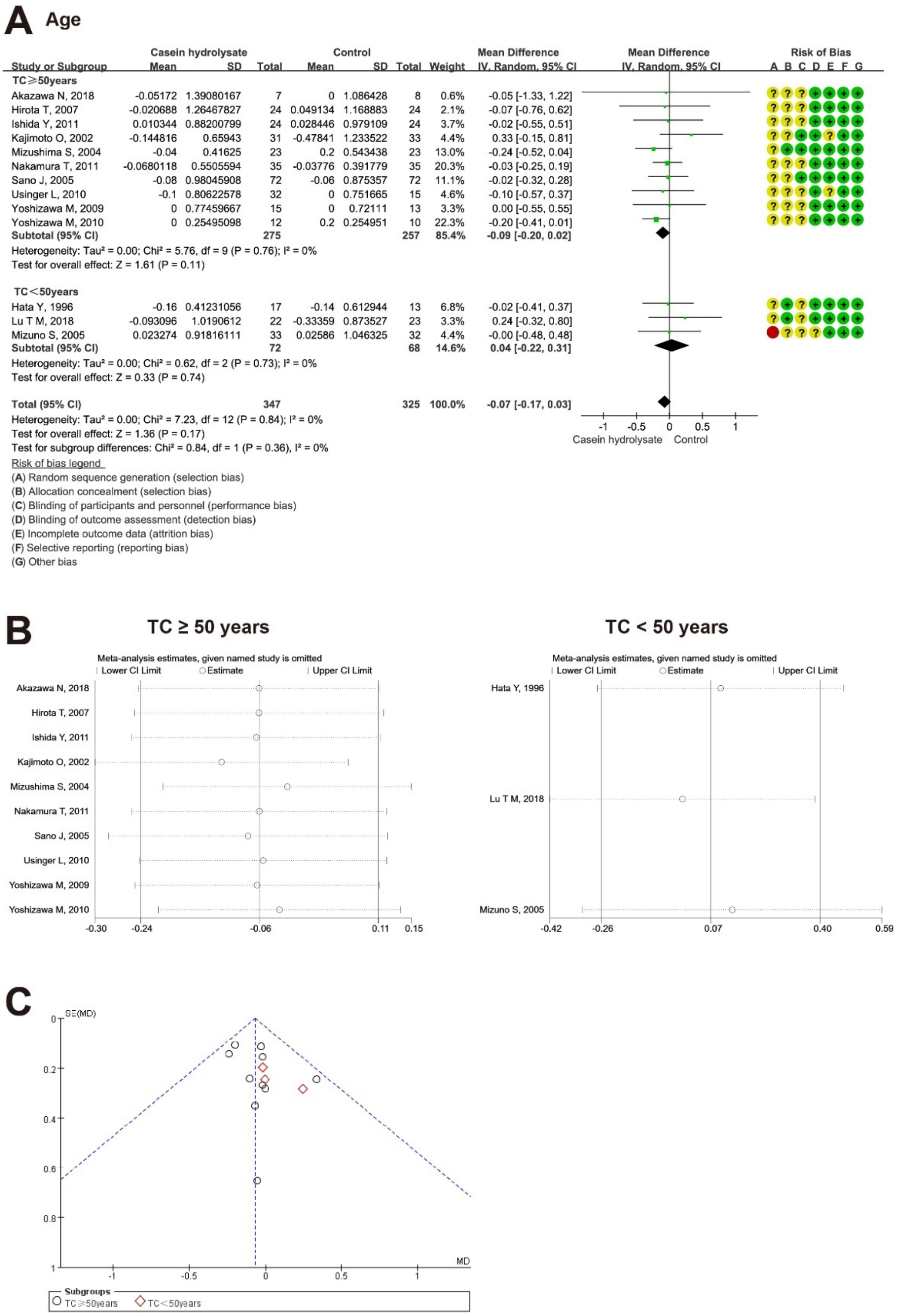

Figure S13. Forest plots (A), sensitivity analysis (B), and funnel plots (C) on TC (mmol/L) in the age analysis. The results represented the changes of TC in the age subgroup with the casein hydrolysate intervention in comparison with the control diet.

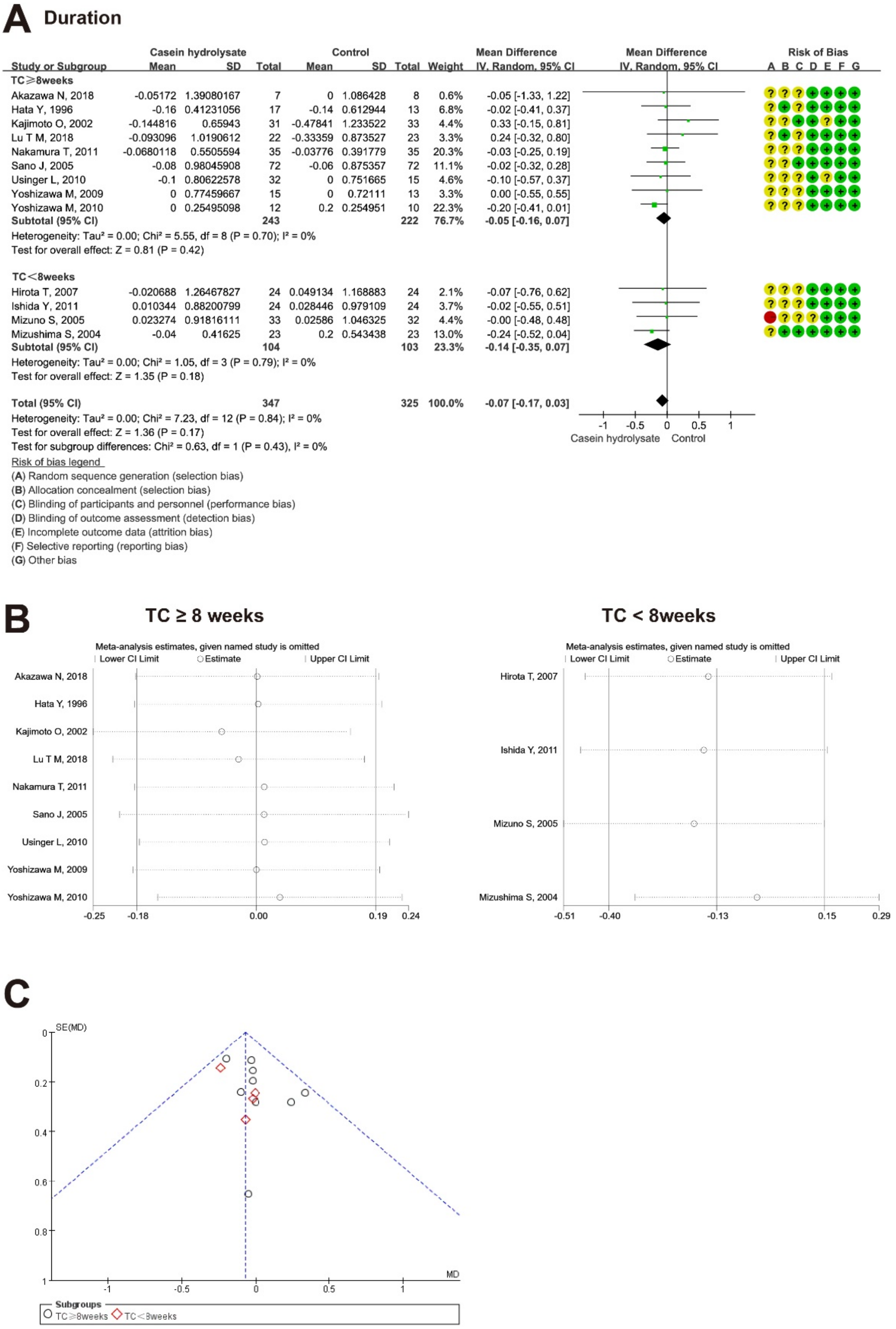

Figure S14. Forest plots (A), sensitivity analysis (B), and funnel plots (C) on TC (mmol/L) in the duration analysis. The results represented the changes of TC in the duration subgroup with the casein hydrolysate intervention in comparison with the control diet.

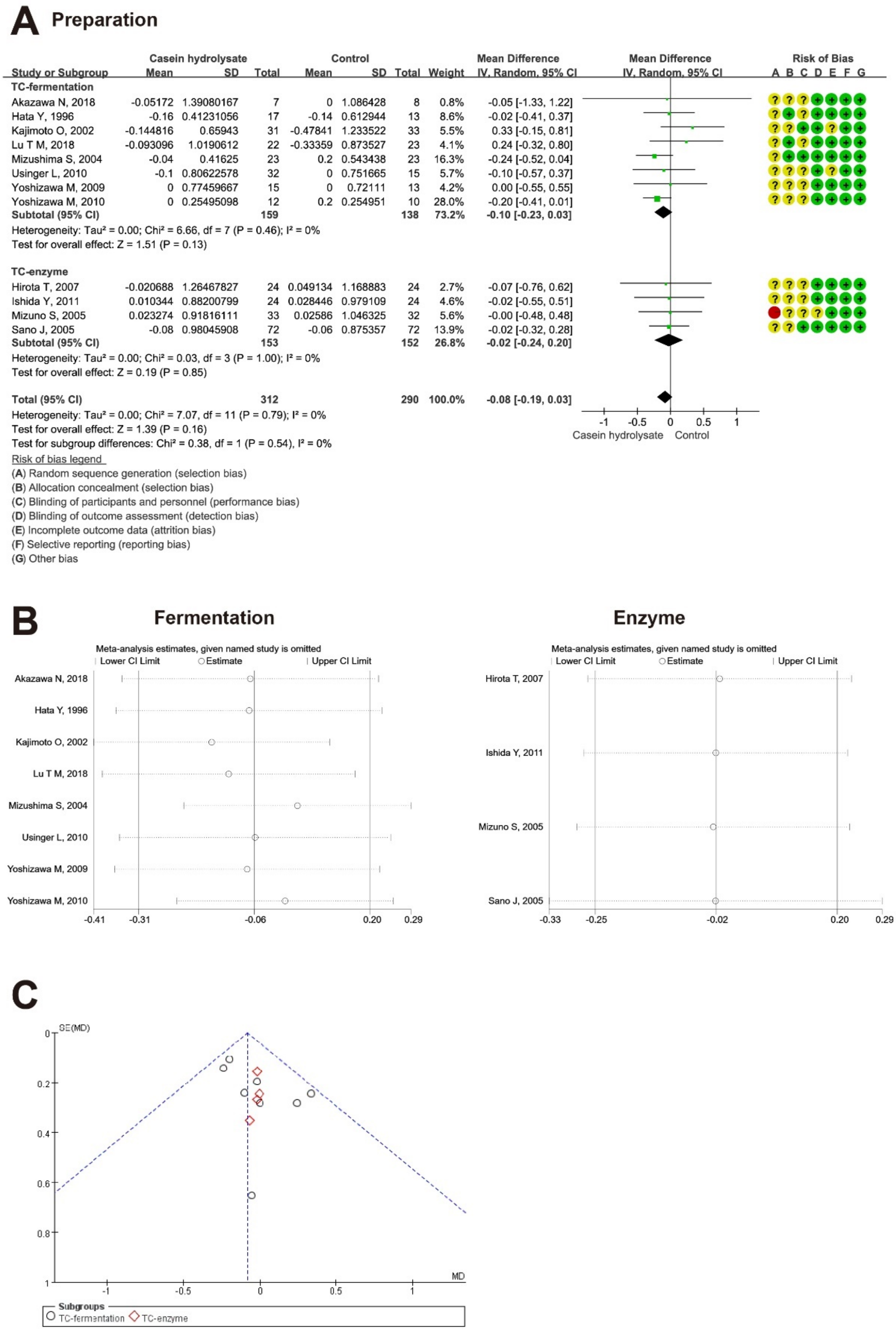

Figure S15. Forest plots (A), sensitivity analysis (B), and funnel plots (C) on TC (mmol/L) in the preparation analysis. The results represented the changes of TC in the preparation subgroup with the casein hydrolysate intervention in comparison with the control diet.

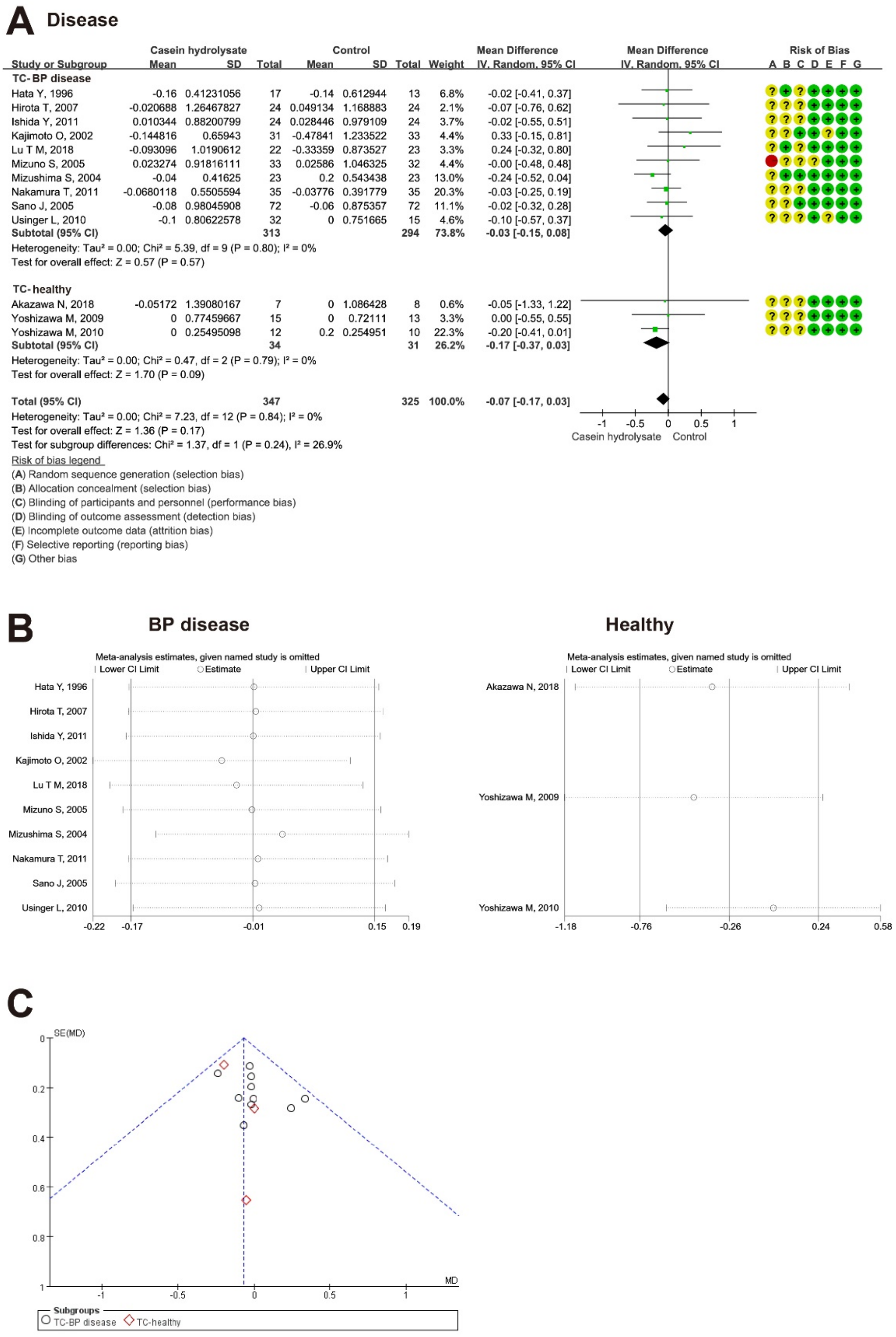

Figure S16. Forest plots (A), sensitivity analysis (B), and funnel plots (C) on TC (mmol/L) in the disease analysis. The results represented the changes of TC in the disease subgroup with the casein hydrolysate intervention in comparison with the control diet.

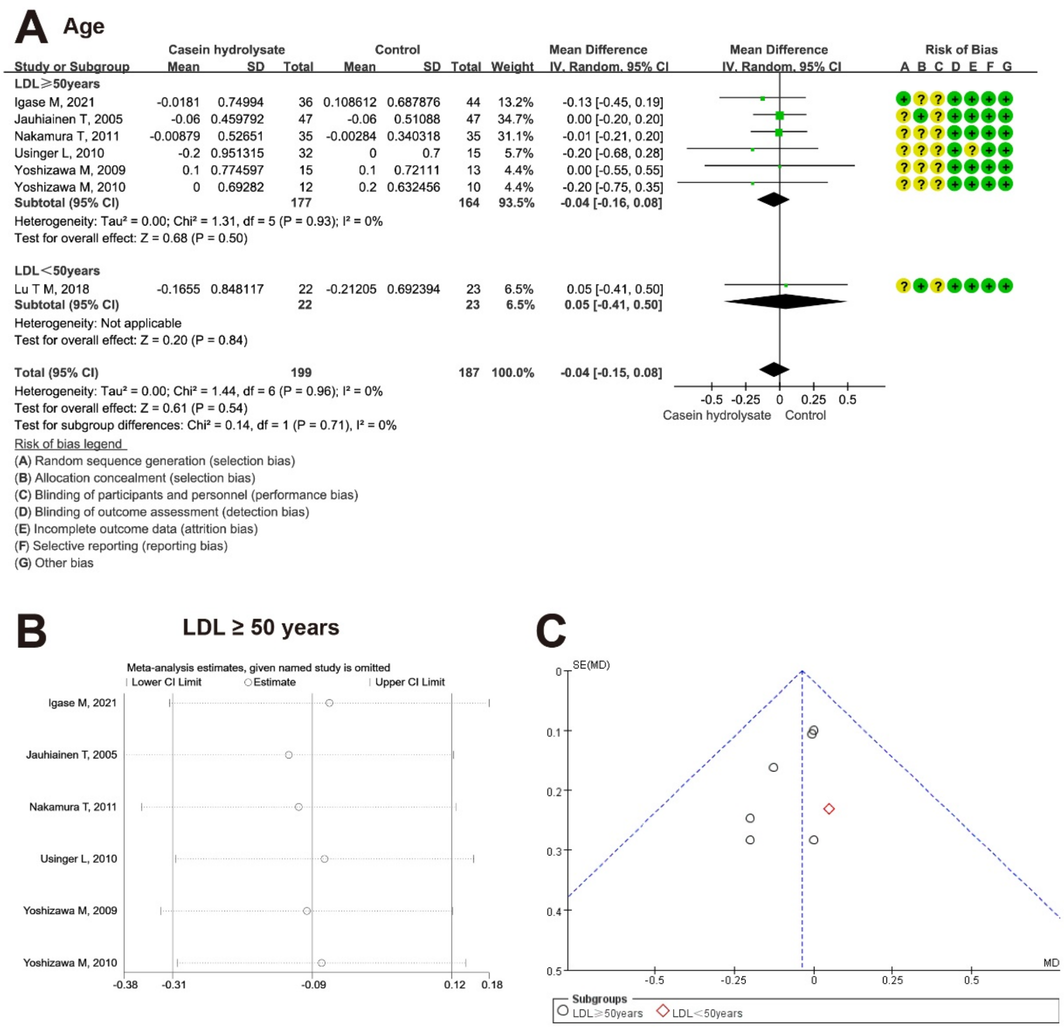

Figure S17. Forest plots (A), sensitivity analysis (B), and funnel plots (C) on LDL (mmol/L) in the age analysis. The results represented the changes of LDL in the age subgroup with the casein hydrolysate intervention in comparison with the control diet.

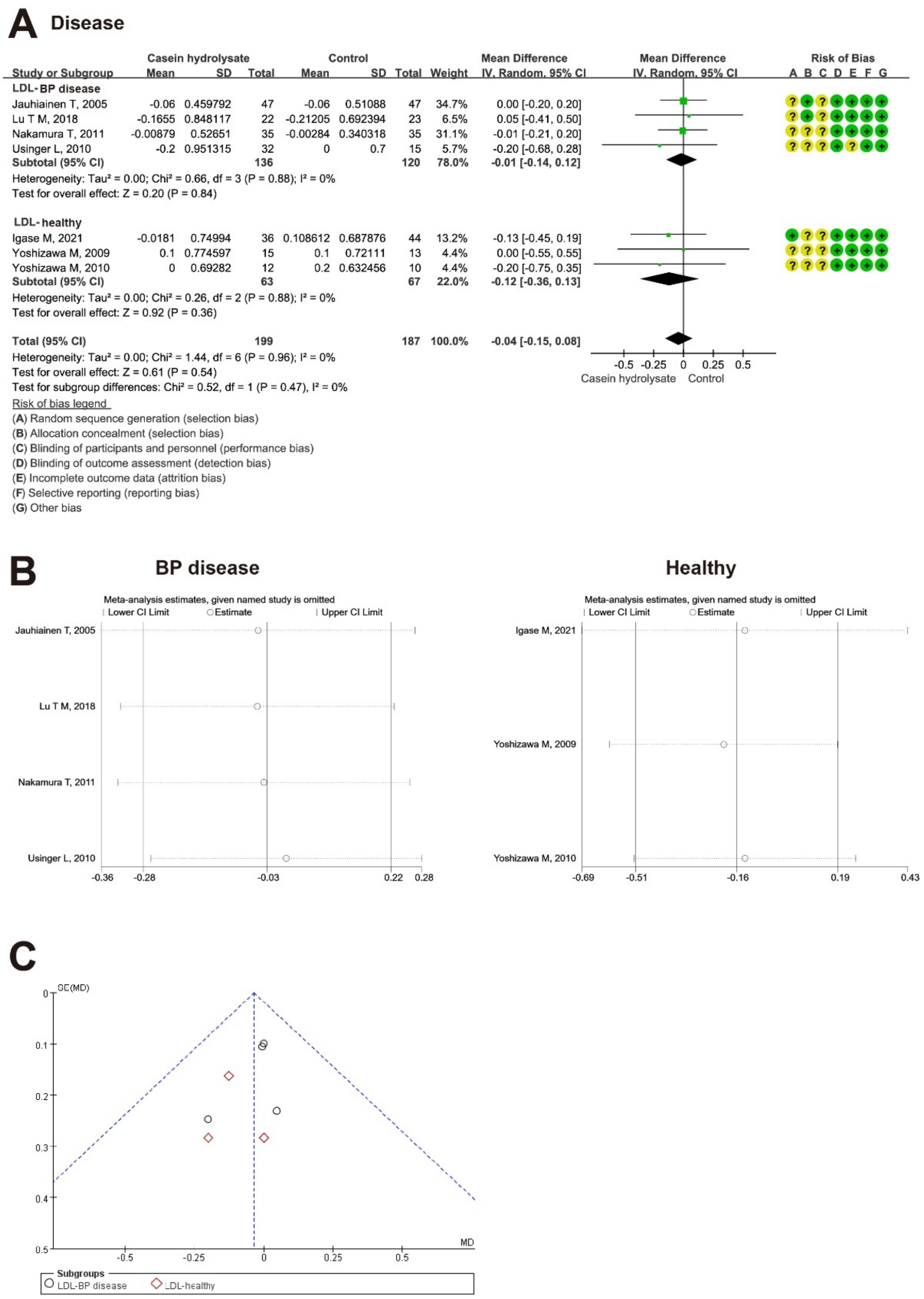

Figure S18. Forest plots (A), sensitivity analysis (B), and funnel plots (C) on LDL (mmol/L) in the disease analysis. The results represented the changes of LDL in the disease subgroup with the casein hydrolysate intervention in comparison with the control diet.

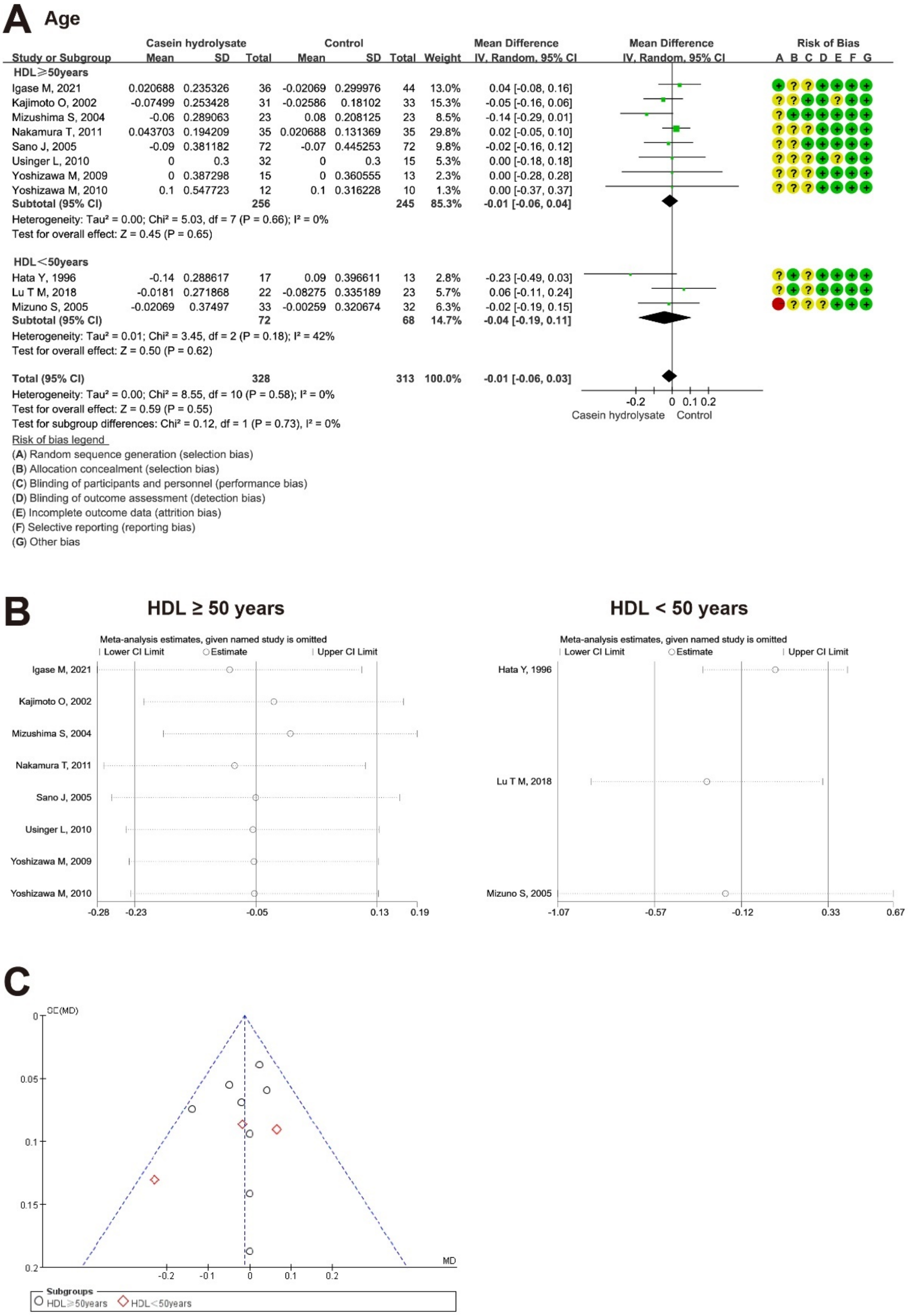

Study or Subgroup	Casein hydrolysate Mean	SD	Total	Control Mean	SD	Total	Weight	Mean Difference IV, Random, 95% CI
**HDL≥50years**								
Igase M, 2021	0.020688	0.235326	36	-0.02069	0.299976	44	13.0%	0.04 [-0.08, 0.16]
Kajimoto O, 2002	-0.07499	0.253428	31	-0.02586	0.18102	33	15.3%	-0.05 [-0.16, 0.06]
Mizushima S, 2004	-0.06	0.289063	23	0.08	0.208125	23	8.5%	-0.14 [-0.29, 0.01]
Nakamura T, 2011	0.043703	0.194209	35	0.020688	0.131369	35	29.8%	0.02 [-0.05, 0.10]
Sano J, 2005	-0.09	0.381182	72	-0.07	0.445253	72	9.8%	-0.02 [-0.16, 0.12]
Usinger L, 2010	0	0.3	32	0	0.3	15	5.3%	0.00 [-0.18, 0.18]
Yoshizawa M, 2009	0	0.387298	15	0	0.360555	13	2.3%	0.00 [-0.28, 0.28]
Yoshizawa M, 2010	0.1	0.547723	12	0.1	0.316228	10	1.3%	0.00 [-0.37, 0.37]
**Subtotal (95% CI)**			256			245	85.3%	-0.01 [-0.06, 0.04]
**HDL<50years**								
Hata Y, 1996	-0.14	0.288617	17	0.09	0.396611	13	2.8%	-0.23 [-0.49, 0.03]
Lu T M, 2018	-0.0181	0.271868	22	-0.08275	0.335189	23	5.7%	0.06 [-0.11, 0.24]
Mizuno S, 2005	-0.02069	0.37497	33	-0.00259	0.320674	32	6.3%	-0.02 [-0.19, 0.15]
**Subtotal (95% CI)**			72			68	14.7%	-0.04 [-0.19, 0.11]
**Total (95% CI)**			328			313	100.0%	-0.01 [-0.06, 0.03]

Figure S19. Forest plots (A), sensitivity analysis (B), and funnel plots (C) on HDL (mmol/L) in the age analysis. The results represented the changes of HDL in the age subgroup with the casein hydrolysate intervention in comparison with the control diet.

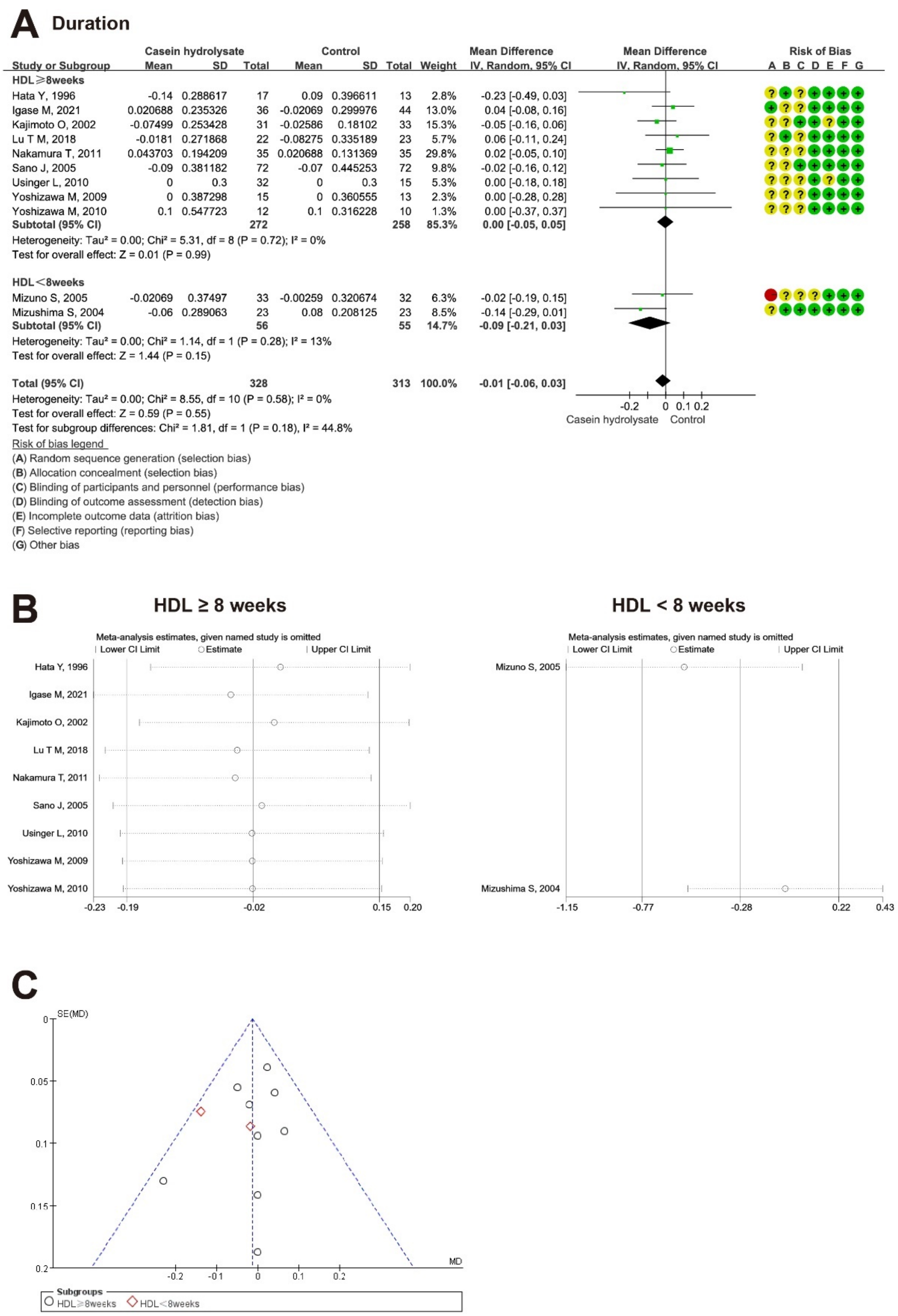

Figure S20. Forest plots (A), sensitivity analysis (B), and funnel plots (C) on HDL (mmol/L) in the duration analysis. The results represented the changes of HDL in the duration subgroup with the casein hydrolysate intervention in comparison with the control diet.

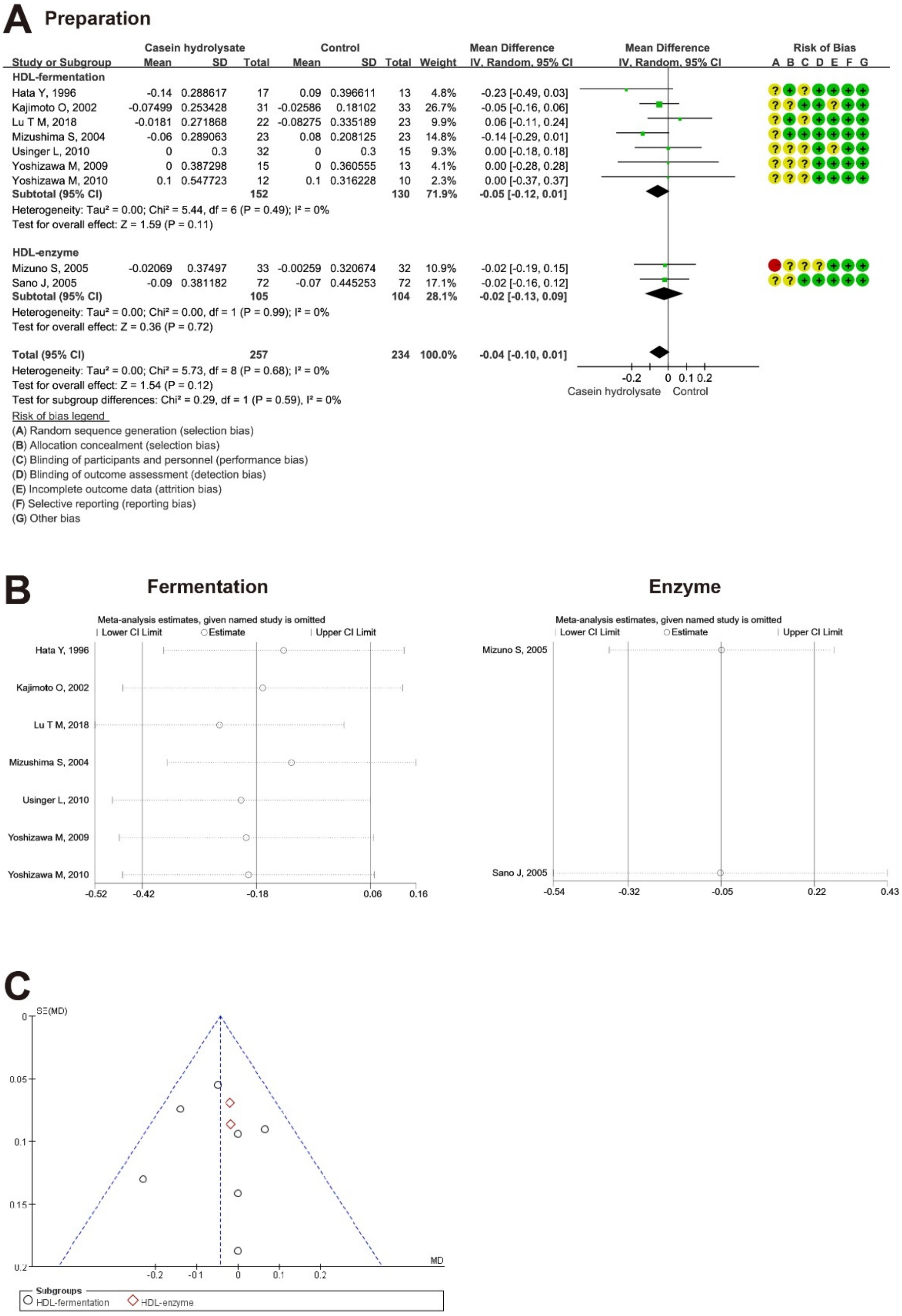

Figure S21. Forest plots (A), sensitivity analysis (B), and funnel plots (C) on HDL (mmol/L) in the preparation analysis. The results represented the changes of HDL in the preparation subgroup with the casein hydrolysate intervention in comparison with the control diet.

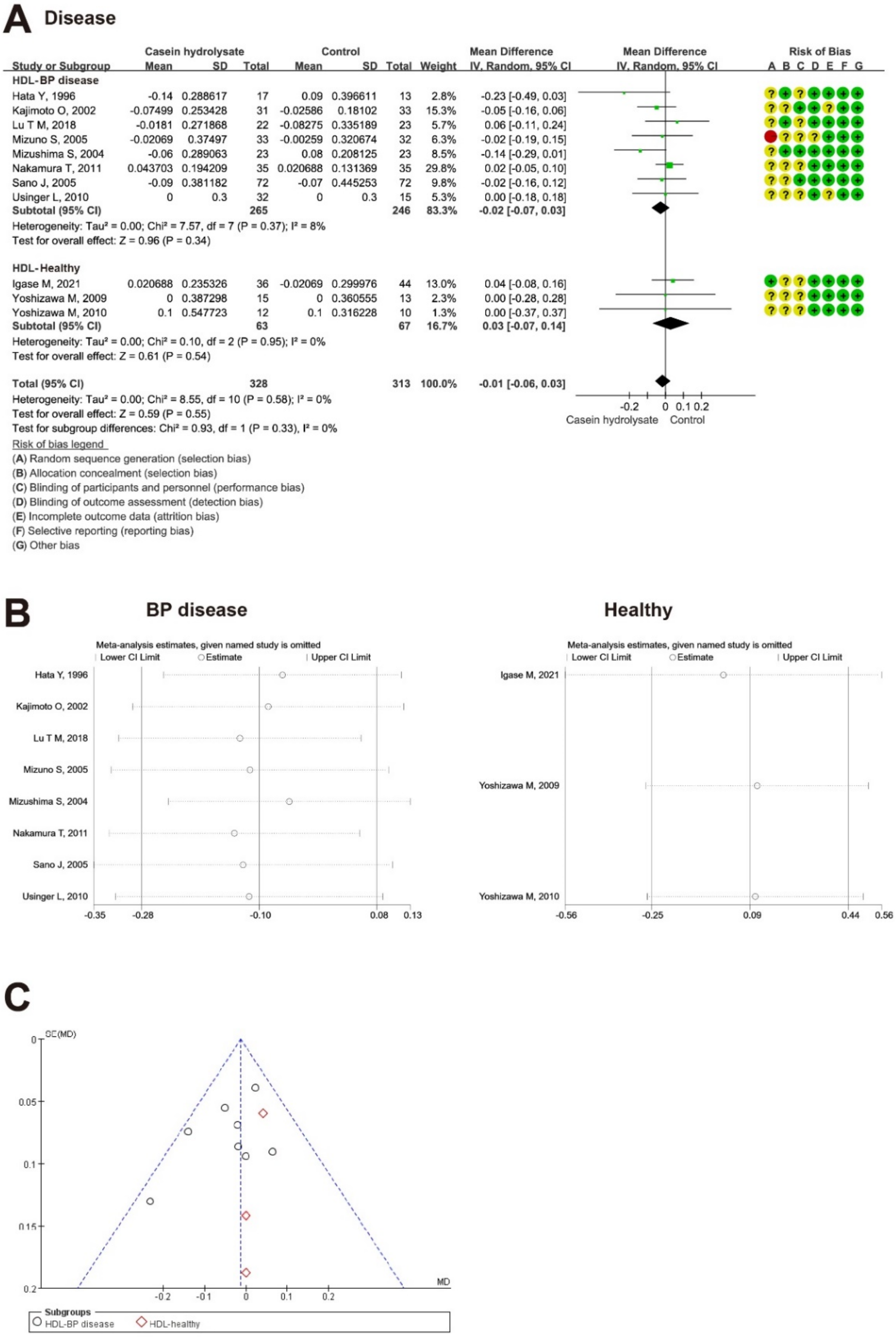

Figure S22. Forest plots (A), sensitivity analysis (B), and funnel plots (C) on HDL (mmol/L) in the disease analysis. The results represented the changes of HDL in the disease subgroup with the casein hydrolysate intervention in comparison with the control diet.

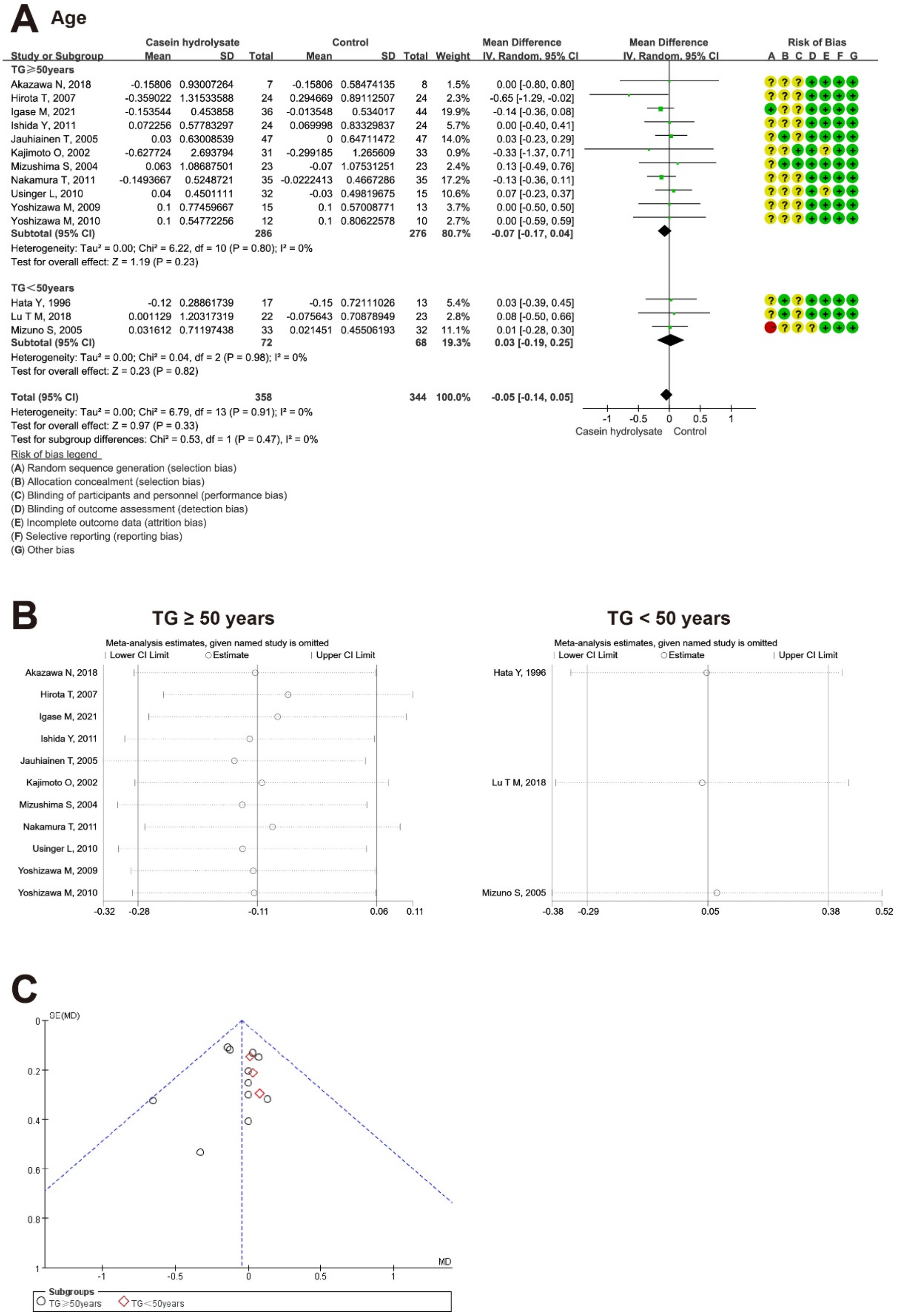

Figure S23. Forest plots (A), sensitivity analysis (B), and funnel plots (C) on TG (mmol/L) in the age analysis**.** The results represented the changes of TG in the age subgroup with the casein hydrolysate intervention in comparison with the control diet.

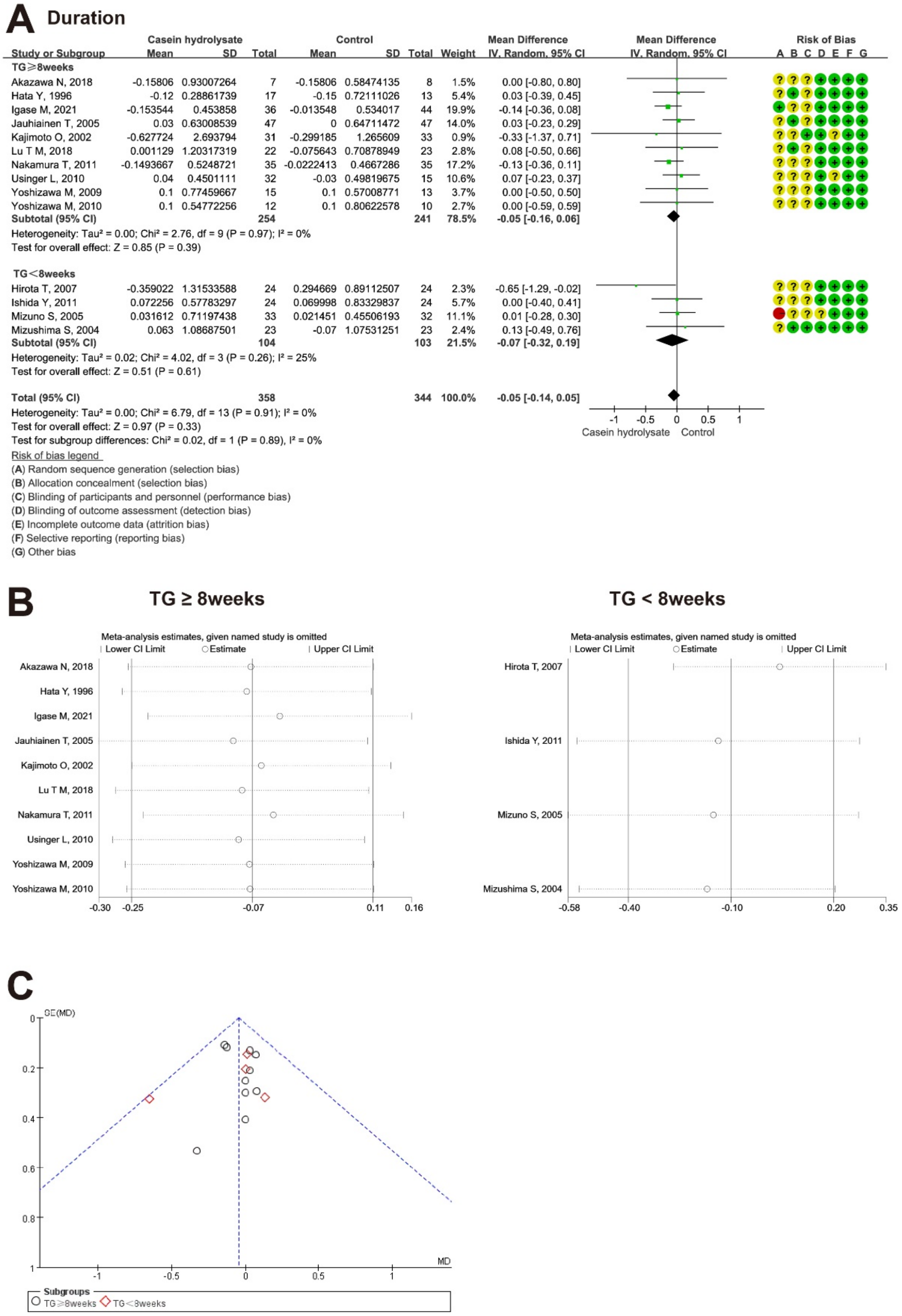

Figure S24. Forest plots (A), sensitivity analysis (B), and funnel plots (C) on TG (mmol/L) in the duration analysis. The results represented the changes of TG in the duration subgroup with the casein hydrolysate intervention in comparison with the control diet.

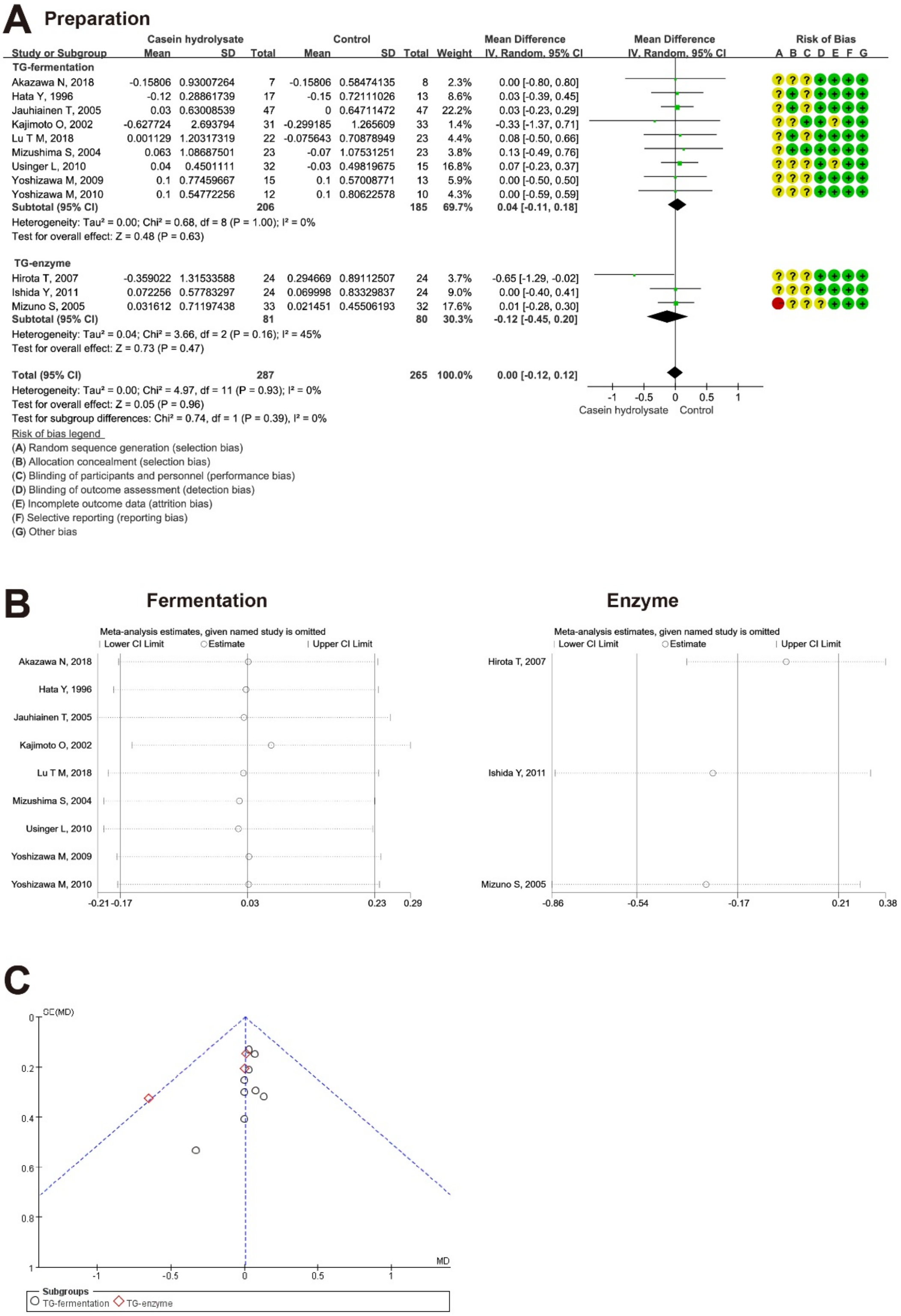

Figure S25. Forest plots (A), sensitivity analysis (B), and funnel plots (C) on TG (mmol/L) in the preparation analysis. The results represented the changes of TG in the preparation subgroup with the casein hydrolysate intervention in comparison with the control diet.

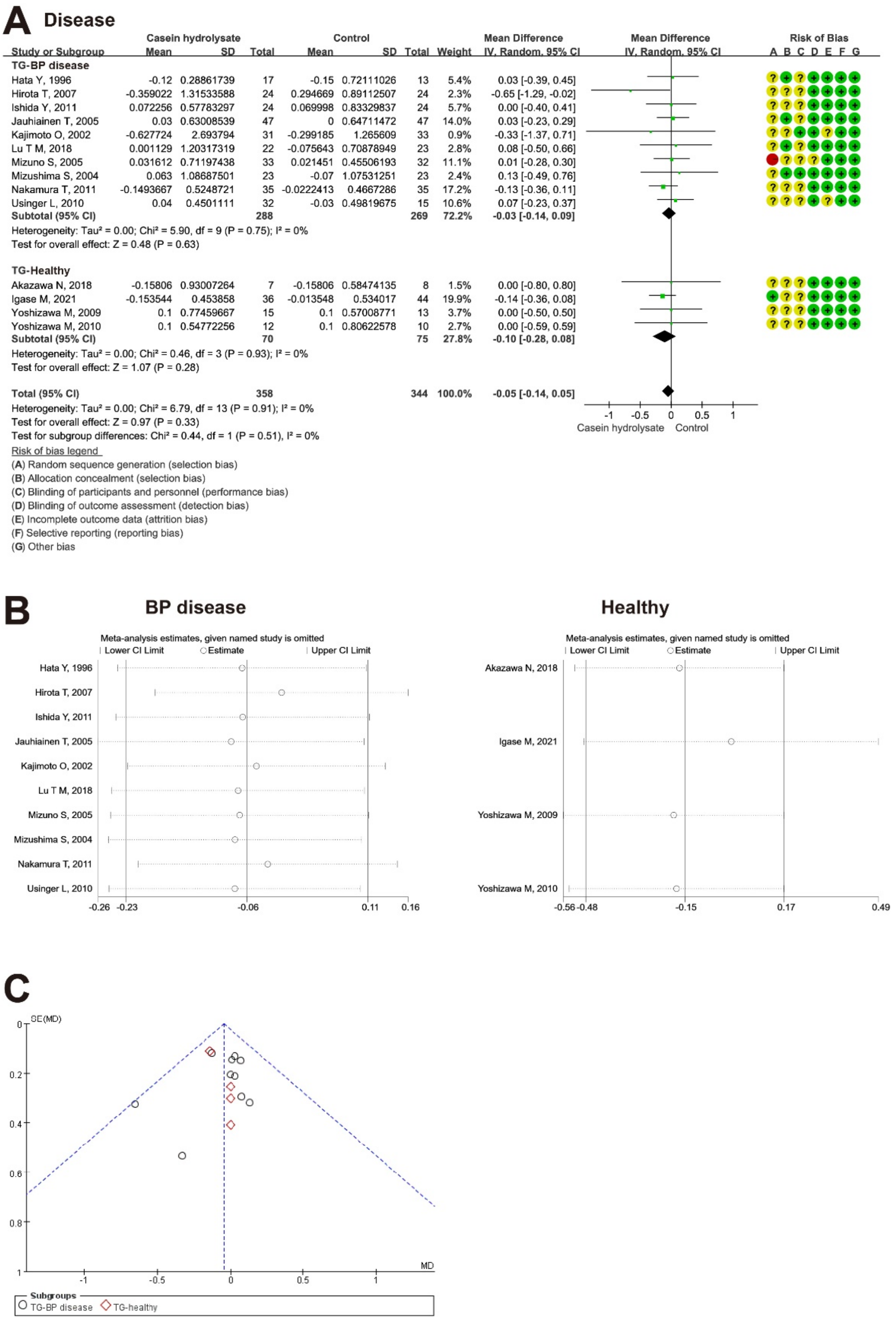

Figure S26. Forest plots (A), sensitivity analysis (B), and funnel plots (C) on TG (mmol/L) in the disease analysis. The results represented the changes of TG in the disease subgroup with the casein hydrolysate intervention in comparison with the control diet.

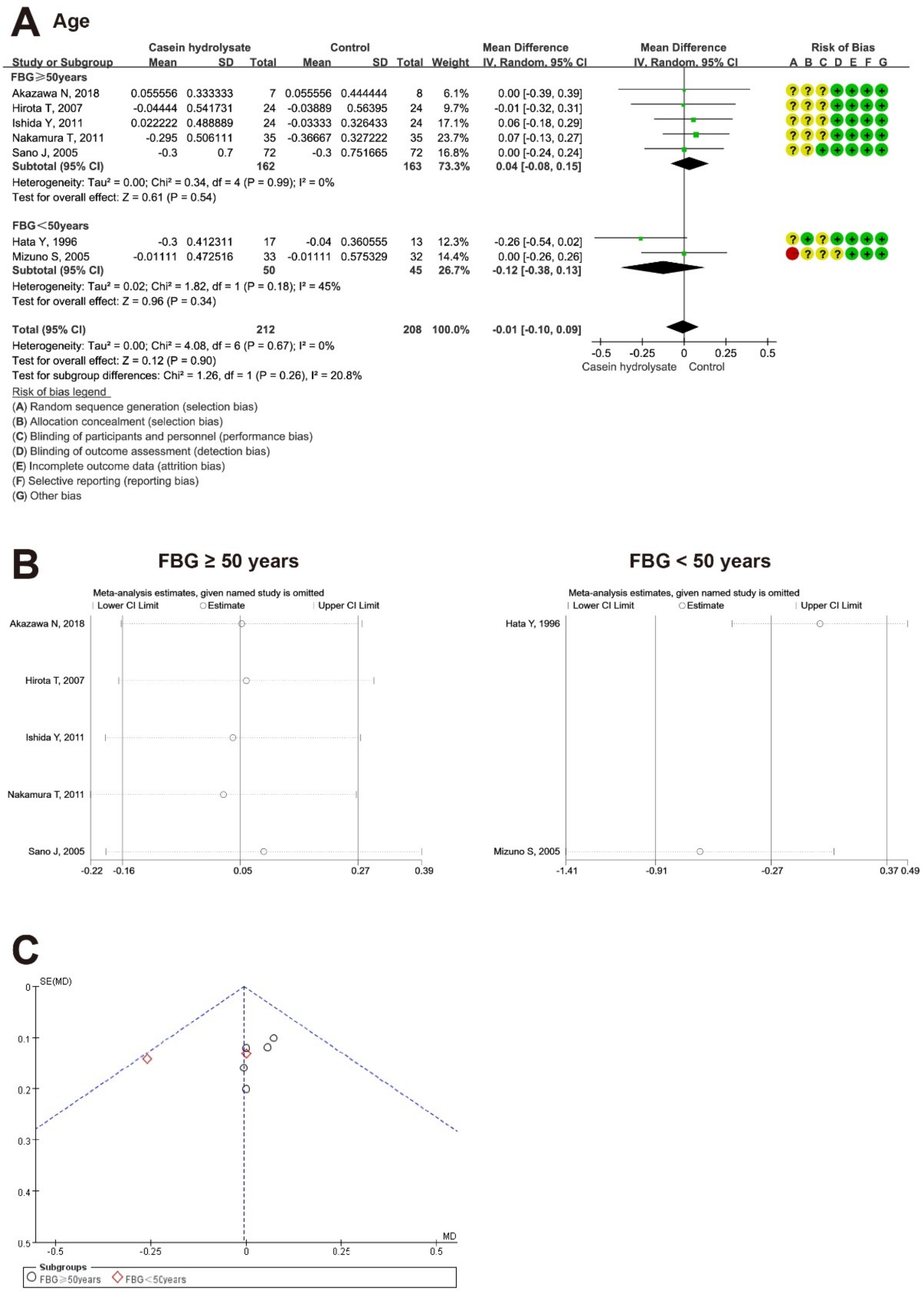

Figure S27. Forest plots (A), sensitivity analysis (B), and funnel plots (C) on FBG (mmol/L) in the age analysis. The results represented the changes of FBG in the age subgroup with the casein hydrolysate intervention in comparison with the control diet.

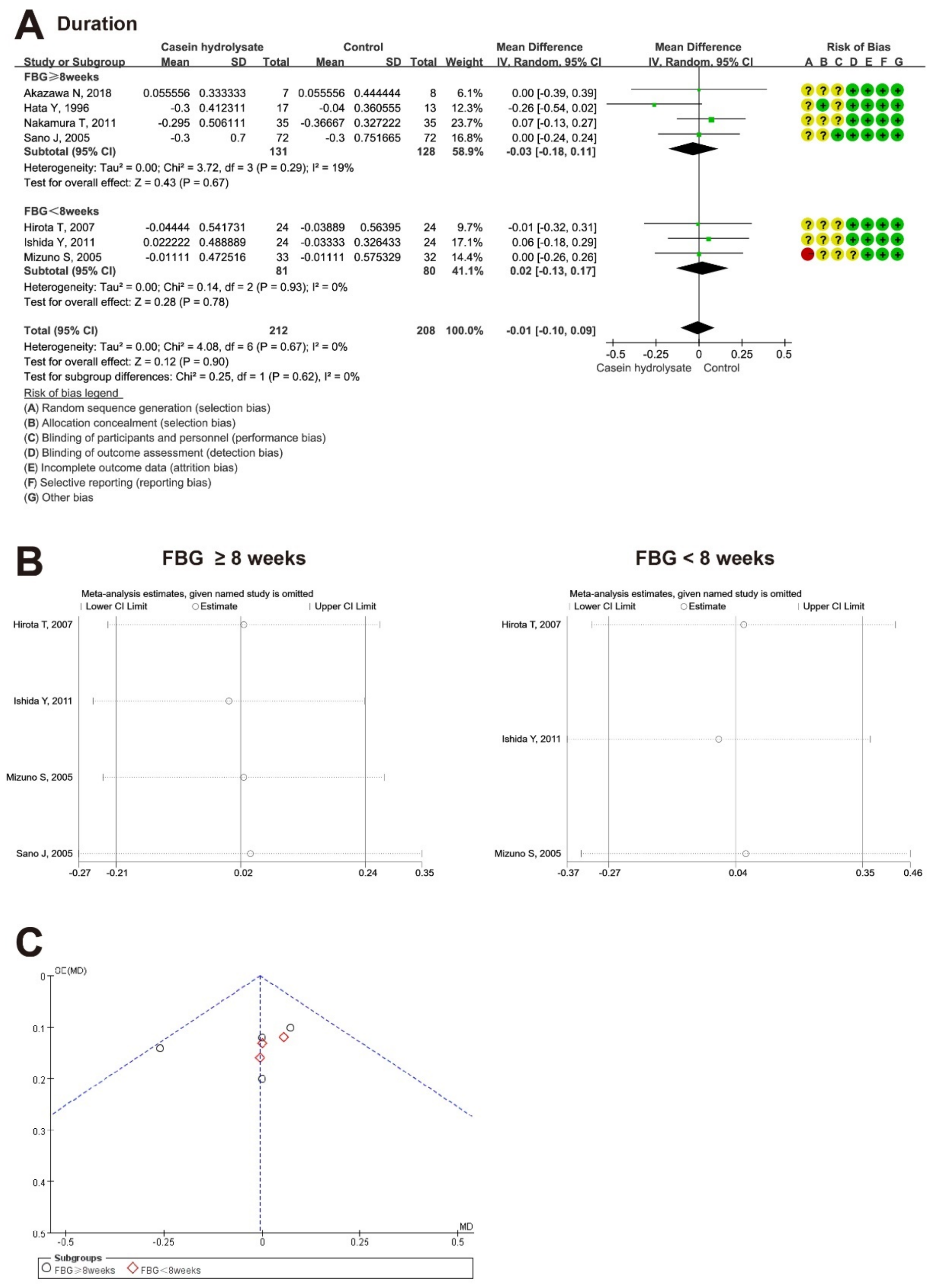

Figure S28. Forest plots (A), sensitivity analysis (B), and funnel plots (C) on FBG (mmol/L) in the duration analysis. The results represented the changes of FBG in the duration subgroup with the casein hydrolysate intervention in comparison with the control diet.

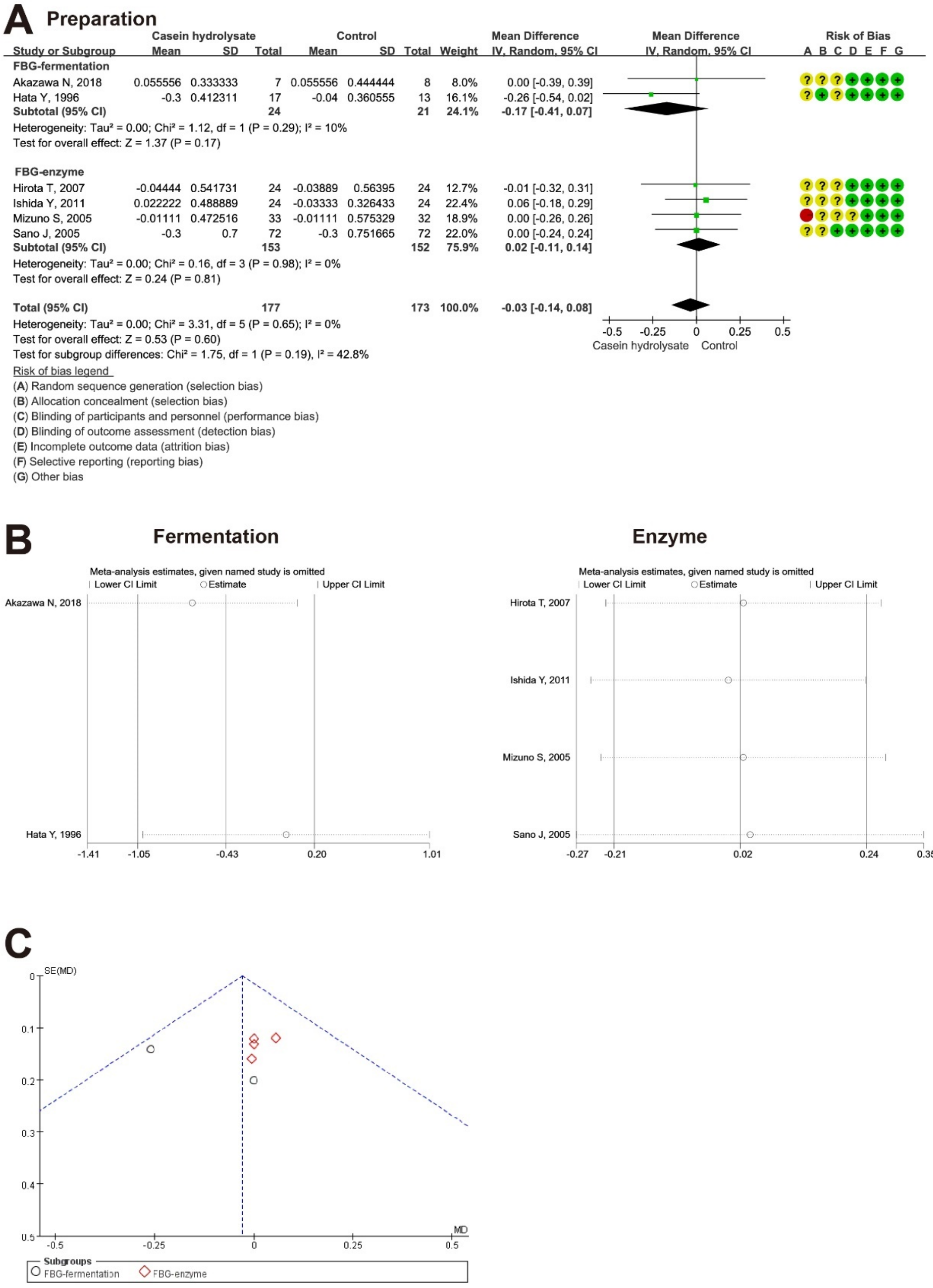

Figure S29. Forest plots (A), sensitivity analysis (B), and funnel plots (C) on FBG (mmol/L) in the preparation analysis. The results represented the changes of FBG in the preparation subgroup with the casein hydrolysate intervention in comparison with the control diet.

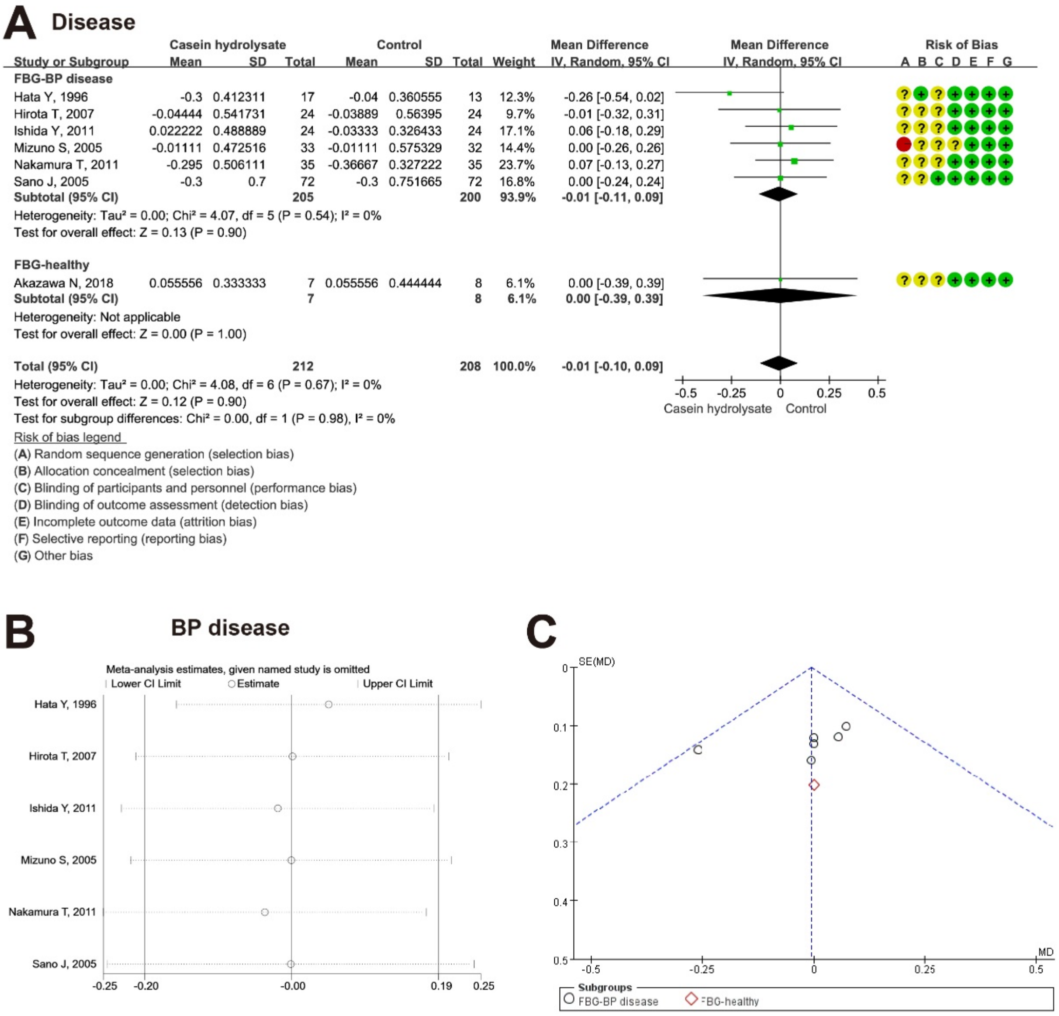

Figure S30. Forest plots (A), sensitivity analysis (B), and funnel plots (C) on FBG (mmol/L) in the disease analysis. The results represented the changes of FBG in the disease subgroup with the casein hydrolysate intervention in comparison with the control diet.
